# Association between preoperative lipid profile and early postoperative objective optical quality after cataract surgery

**DOI:** 10.3389/fendo.2026.1887440

**Published:** 2026-10-09

**Authors:** Huaixiao Zang, Yuxin Liu, Dong Shi

**Affiliations:** 1 Department of Ophthalmology, The Fourth Affiliated Hospital of China Medical University, Shenyang, China; 2 Eye Hospital of China Medical University, Shenyang, China

**Keywords:** cataract surgery, lipid profile, low-density lipoprotein cholesterol, optical quality, phacoemulsification, visual quality

## Abstract

**Objective:**

This study examined the association between preoperative lipid levels and early postoperative objective visual quality after cataract surgery.

**Methods:**

This retrospective study included patients who underwent phacoemulsification with implantation of the same monofocal intraocular lens (IOL) model between November 2024 and May 2025. Preoperative serum total cholesterol (TC), triglycerides (TG), high-density lipoprotein cholesterol (HDL-C), and low-density lipoprotein cholesterol (LDL-C) levels were recorded. Patients were grouped according to guideline-based lipid criteria. Objective visual quality was evaluated at postoperative day 1, week 1, and month 1 using the iTrace aberrometer and OQAS II system. Outcomes included best-corrected visual acuity (BCVA), dysfunctional lens index (DLI), objective scatter index (OSI), higher-order aberrations (HOAs), modulation transfer function (MTF), and Strehl ratio (SR). Between-group comparisons, Spearman correlation analysis, multivariable regression, and adjusted linear mixed-effects models were performed.

**Results:**

The final analysis included 110 eyes from 110 patients. BCVA and objective optical quality improved during the first postoperative month. During follow-up, DLI, MTF, and SR increased, whereas OSI and HO total decreased. At postoperative month 1, the above-target LDL-C group showed higher OSI and HO Total values and lower MTF and SR values than the at-target group, while BCVA did not differ significantly. After adjustment for potential confounders, above-target LDL-C remained associated with higher HO Total (β = 0.121, 95% CI, 0.034 to 0.207; FDR-adjusted P = 0.049) and lower SR Internal (β = -0.050, 95% CI, -0.086 to -0.014; FDR-adjusted P = 0.049). Correlation analyses also showed associations of TC, TG, and LDL-C with several optical quality measures, whereas no clear association was observed for HDL-C. In the adjusted longitudinal models, LDL-C-related associations were more limited, and the association with BCVA was strongest at postoperative day 1 and attenuated over time.

**Conclusion:**

Preoperative lipid status may be related to early postoperative objective visual quality after cataract surgery. The clearest differences were observed for LDL-C at postoperative month 1. Further prospective studies are needed to confirm this relationship and clarify its clinical significance.

## 1 Introduction

Cataract surgery has evolved from a procedure primarily focused on vision restoration to one that places growing emphasis on postoperative visual quality (1). With advances in surgical techniques and IOL design, patients now expect clearer vision together with better overall visual performance (2). Consequently, objective optical quality parameters are being used more widely to enable a more comprehensive assessment after cataract surgery (3).

Several factors can affect visual quality after cataract surgery. Some are related to the surgical procedure itself. Others occur during the postoperative period, including ocular surface disturbance, tear film instability, and inflammatory responses (4). During early recovery, even small changes in the tear film or ocular surface may affect optical quality (5, 6). Systemic metabolic status may also contribute to this recovery process. Dyslipidemia is a common metabolic abnormality and has been associated with tear-film changes, meibomian gland dysfunction, and dry eye disease (7, 8). Altered lipid metabolism is also closely related to low-grade systemic inflammation, providing another possible link between systemic metabolic status and the postoperative ocular environment (9). Together, these observations suggest that preoperative lipid status may be relevant to early postoperative optical recovery after cataract surgery.

In clinical practice, TC, TG, HDL-C, and LDL-C are routinely measured lipid parameters. However, little is known about whether these common metabolic markers are associated with early postoperative optical quality after cataract surgery. Therefore, this study aimed to investigate the associations of preoperative TC, TG, HDL-C, and LDL-C levels with early postoperative objective visual quality after cataract surgery.

## 2 Methods

### 2.1 Study population

This single-center retrospective study was conducted at the Department of Ophthalmology, the Fourth Affiliated Hospital of China Medical University (Shenyang, China). Patients who underwent phacoemulsification with monofocal IOL implantation between November 2024 and May 2025 were screened for eligibility. Only one eye per patient was included. In total, 120 eyes from 120 patients were initially reviewed.

The inclusion criteria were as follows: 1) age 50–80 years; 2) age-related cataract scheduled for phacoemulsification; 3) axial length of 22.0–26.0 mm, corneal astigmatism < 1.5 diopters, and corneal endothelial cell density > 2000 cells/mm²; 4) normal pupillary function. The axial-length range of 22.0–26.0 mm was selected because IOL power calculation is less predictable in short and long eyes (10). Corneal astigmatism was limited to <1.5 D because higher preoperative astigmatism may increase postoperative residual astigmatism and often requires specific astigmatic correction strategies (11, 12). An endothelial cell density >2000 cells/mm² was required to minimize the potential influence of reduced endothelial reserve and postoperative corneal edema (13). The exclusion criteria were as follows: 1) ocular disorders other than cataract (including high myopia, amblyopia, severe ocular surface disease, glaucoma, corneal or retinal pathology, or optic nerve disorders); 2) a history of ocular trauma, inflammation, or surgery (including refractive or intraocular procedures); 3) systemic conditions that might affect visual quality assessment or postoperative recovery; 4) a history of atherosclerotic cardiovascular disease (ASCVD), including coronary heart disease, or chronic kidney disease (CKD); 5) prior use of lipid-lowering medication. After screening, 10 patients were excluded, including 2 with ASCVD, coronary heart disease, or CKD, 3 with prior lipid-lowering therapy, 4 with ocular disorders other than cataract, and 1 with systemic conditions that might affect visual quality assessment or postoperative recovery. After these exclusions, 110 eyes from 110 patients remained for analysis. Patients with established ASCVD, CKD, or previous lipid-lowering treatment were excluded to limit potential confounding related to baseline lipid status and postoperative outcomes. All included patients belonged to the ASCVD primary prevention population. The patient selection process is shown in **Figure 1**. As this was a retrospective study, no *a priori* sample size calculation was performed. All eligible patients who met the predefined inclusion and exclusion criteria during the study period were included in the analysis.

**Figure 1:**
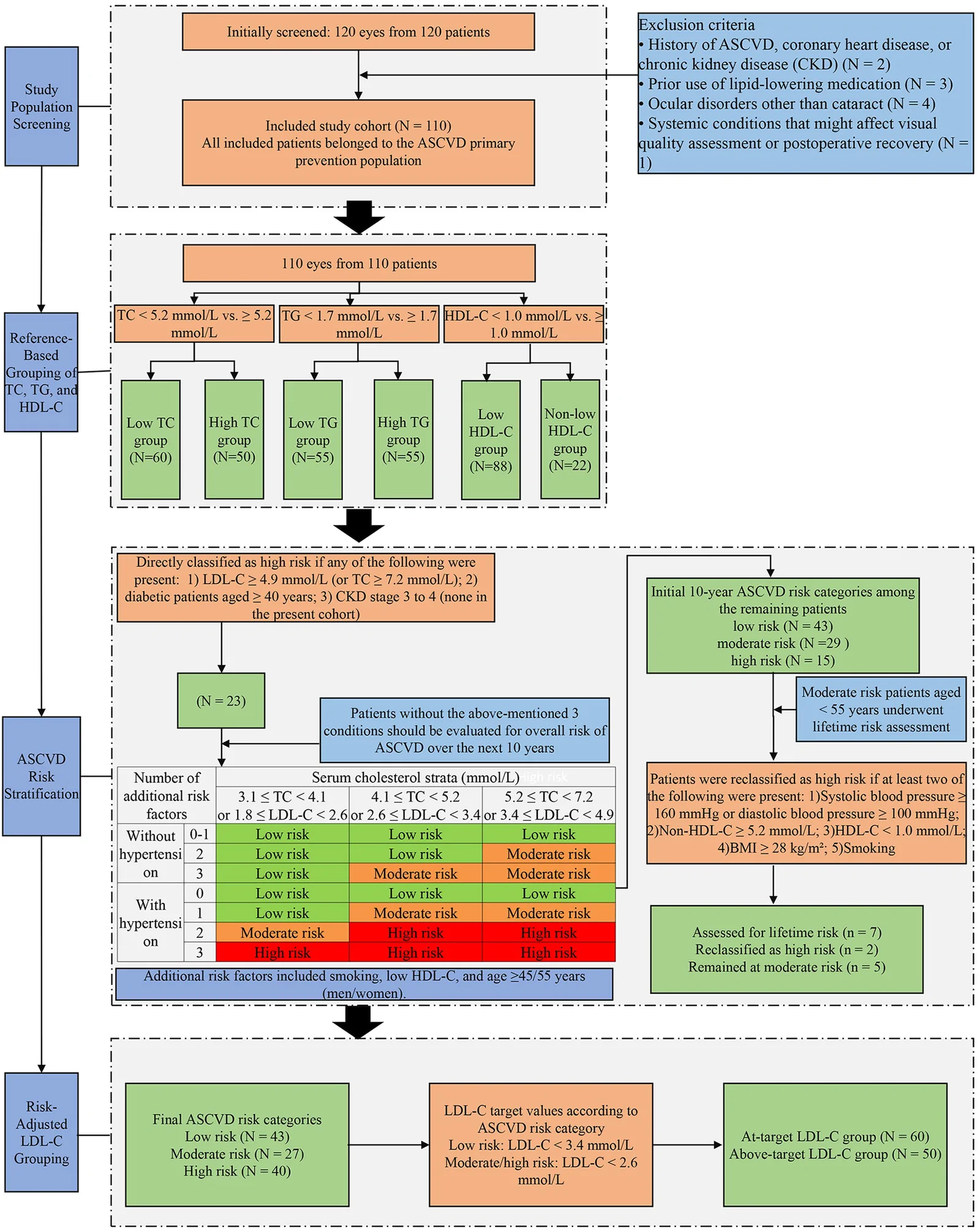
Flowchart of patient selection. ASCVD, atherosclerotic cardiovascular disease; HDL-C, high-density lipoprotein cholesterol; LDL-C, low-density lipoprotein cholesterol; TC, total cholesterol; TG, triglycerides. After screening, 110 eyes from 110 patients were included, all of whom belonged to the ASCVD primary prevention population. TC, TG, and HDL-C were grouped using fixed reference cutoffs, whereas LDL-C was grouped according to ASCVD risk-specific target levels based on the 2023 China Guidelines for Lipid Management. ASCVD risk stratification included direct classification of high-risk individuals, 10-year ASCVD risk assessment, and lifetime ASCVD risk assessment in moderate-risk participants aged <55 years.

The study protocol received approval from the Ethics Committee of the Fourth Affiliated Hospital of China Medical University (EC-2026-KS-098). All participants provided written informed consent.

### 2.2 Data collection and visual quality assessment

Clinical and laboratory data were retrospectively extracted from the medical records. Baseline data covered demographic characteristics, systemic comorbidities, and ocular parameters, including age, sex, body mass index (BMI), marital status, smoking history, diabetes, hypertension, operated eye, BCVA, axial length (AL), corneal astigmatism, corneal endothelial cell density (CEC), and IOL power. Serum lipid levels were measured using fasting venous blood samples obtained on the day before surgery after at least 12 hours of fasting. The assessed lipid parameters included TC, TG, HDL-C, and LDL-C.

Preoperative ophthalmic evaluation included BCVA, slit-lamp biomicroscopy, intraocular pressure measurement, and corneal topography (Pentacam; Oculus, Wetzlar, Germany). Biometric parameters, including AL, anterior chamber depth (ACD), and corneal curvature, were measured using the IOLMaster (Carl Zeiss Meditec, Jena, Germany). Fundus status was assessed using scanning laser ophthalmoscopy and optical coherence tomography.

Postoperative examinations were performed at 1 day, 1 week, and 1 month after surgery. At each visit, BCVA was recorded at 5 m using a logarithm of the minimum angle of resolution (logMAR) chart. Objective visual quality was assessed using the iTrace aberrometer and the OQAS II system. The iTrace provided HO total, DLI, MTF, and SR (14). MTF values were recorded at spatial frequencies of 5, 10, 15, 20, 25, and 30 cycles per degree. MTF and SR were further analyzed for the total-eye, internal, and corneal components to distinguish anterior segment and intraocular contributions to optical quality. The OQAS II system was used to measure the OSI (15). During testing, spherical error was automatically compensated by the device, whereas cylindrical error was partially corrected with external trial lenses. All optical quality measurements were obtained at a pupil diameter of 3.0 mm. To reduce measurement variability, all examinations were performed in the same examination room by the same group of experienced technicians using identical instrument settings. Intraoperative and postoperative complications documented during the 1-month follow-up were also recorded.

### 2.3 Surgical procedure and perioperative medication

All patients followed the same perioperative management protocol. Topical gatifloxacin was started 1 day before surgery and administered four times daily. On the day of surgery, pupils were dilated with 0.5% compound tropicamide eye drops until adequate mydriasis was achieved. Topical anesthesia was induced with 0.5% proparacaine hydrochloride.

All procedures were performed by the same experienced surgeon using a standard phacoemulsification technique. A 3.0 mm clear corneal incision was created at a location determined by preoperative corneal topography. After continuous curvilinear capsulorhexis, the lens nucleus was removed by phacoemulsification. All patients received the same monofocal IOL model (Rayner RAO600C), which was implanted into the capsular bag using a preloaded injector system. After surgery, all patients received topical gatifloxacin, prednisolone acetate, and recombinant bovine basic fibroblast growth factor. The postoperative regimen was gradually tapered according to the clinical course during follow-up.

### 2.4 ASCVD risk stratification and lipid grouping

All included patients belonged to the ASCVD primary prevention population. ASCVD risk stratification was performed according to the 2023 China Guidelines for Lipid Management (16). Patients were directly classified as high risk if any of the following conditions were present: LDL-C ≥ 4.9 mmol/L (or TC ≥ 7.2 mmol/L), diabetes mellitus in patients aged ≥ 40 years, or CKD stage 3–4. As patients with CKD were excluded at enrollment, no patient met the CKD criterion in the present cohort. For the remaining patients, 10-year ASCVD risk was assessed according to the guideline flow chart based on serum cholesterol strata, history of hypertension, and the cumulative number of additional risk factors. Additional risk factors included smoking history, low HDL-C, and age ≥ 45/55 years in men/women.

Patients with a moderate 10-year ASCVD risk who were younger than 55 years underwent further lifetime ASCVD risk assessment. They were reclassified as high risk if at least two of the following conditions were present: systolic blood pressure ≥ 160 mmHg or diastolic blood pressure ≥ 100 mmHg, non-HDL-C ≥ 5.2 mmol/L, HDL-C < 1.0 mmol/L, BMI ≥ 28 kg/m², and smoking. Non-HDL-C was calculated as TC minus HDL-C. For lifetime risk assessment, the blood pressure criterion was considered present when it was met in at least two of the following three records: documented highest previous blood pressure, blood pressure measured at admission, and blood pressure measured before surgery. Diabetes was defined according to documented medical history. Hypertension history used in the 10-year ASCVD risk assessment was defined according to documented medical history.

TC, TG, and HDL-C were grouped using fixed reference cutoffs from the 2023 China Guidelines for Lipid Management. TC was categorized as < 5.2 mmol/L or ≥ 5.2 mmol/L, TG as < 1.7 mmol/L or ≥ 1.7 mmol/L, and HDL-C as low HDL-C (< 1.0 mmol/L) or non-low HDL-C (≥ 1.0 mmol/L). Because the guideline provides risk-stratified treatment targets specifically for LDL-C, LDL-C grouping was based on ASCVD risk-specific target levels rather than a fixed cutoff. For participants classified as low risk, the LDL-C target was < 3.4 mmol/L, whereas for those classified as moderate or high risk, the LDL-C target was < 2.6 mmol/L. Participants were therefore assigned to either the at-target LDL-C group or the above-target LDL-C group. The overall stratification and grouping process is shown in **Figure 1**.

### 2.5 Statistical analysis

Statistical analyses were conducted using SPSS version 31 (IBM Corp., Armonk, NY, USA) and R version 4.5.2 (R Foundation for Statistical Computing, Vienna, Austria). The normality of continuous variables was checked with the Shapiro–Wilk test. Normally distributed data are presented as mean ± standard deviation (SD), while non-normal data are summarized as median (IQR). Categorical variables are shown as number and percentage.

Baseline characteristics were compared between the corresponding lipid groups. Continuous variables were compared using the independent-samples t test or Mann–Whitney U test. Categorical variables were assessed using the chi-square test or Fisher’s exact test depending on distributional assumptions. For TC, TG, HDL-C, and LDL-C, group-based comparisons of postoperative visual quality were performed at postoperative day 1, week 1, and month 1. These between-group comparisons were not adjusted for covariates, although the corresponding P values were corrected for multiple testing using the Benjamini–Hochberg false discovery rate method. Because LDL-C is the primary lipid target in the current guideline framework, LDL-C-related between-group results are reported in the main text. The corresponding results for TC, TG, and HDL-C are provided in the Supplementary Materials.

As a sensitivity analysis, multivariable linear regression was performed for the major optical quality outcomes to determine whether postoperative month 1 differences between LDL-C target groups remained after adjustment for potential confounders. In each model, the postoperative month 1 outcome was entered as the dependent variable, and LDL-C target group was the primary independent variable, with the at-target group as the reference. The models were adjusted for age, sex, body mass index, smoking status, diabetes, hypertension, preoperative BCVA, axial length, and corneal astigmatism, using the same covariates as in the longitudinal analyses. HC3 heteroscedasticity-consistent standard errors were used to calculate 95% confidence intervals and two-sided P values. P values for the LDL-C group coefficient were adjusted for multiple comparisons using the Benjamini–Hochberg false discovery rate method. An FDR-adjusted P value < 0.05 was considered statistically significant. To assess whether the baseline differences in marital status and IOL power influenced the HDL-C findings, the HDL-C longitudinal models were additionally adjusted for these two variables while retaining the covariates included in the primary longitudinal analyses. P values from these extended models were adjusted using the Benjamini–Hochberg false discovery rate method.

Spearman correlation analysis examined the relationships between preoperative lipid parameters and postoperative month 1 visual quality indices. Correlation coefficients are presented as *r* values with corresponding two-sided *P* values.

Longitudinal changes in postoperative visual outcomes were evaluated using linear mixed-effects models. Patient ID was entered as a random intercept term (1|ID) to account for within-subject correlation across repeated measurements. Two sets of models were constructed. The first model examined overall changes in visual acuity and optical quality over time, with postoperative day 1 as the reference point. The second included separate models for TC, TG, HDL-C, and LDL-C. These models assessed the longitudinal association of one lipid parameter with postoperative visual outcomes. Each model included time, the corresponding lipid parameter, and the interaction between time and that lipid parameter. The interaction terms were used to determine whether the association between each lipid parameter and postoperative outcomes changed across follow-up visits.

The adjusted models included age, sex, body mass index, smoking history, diabetes, hypertension, preoperative best-corrected visual acuity, axial length, and corneal astigmatism as covariates. Regression results are presented as β coefficients with 95% confidence intervals (CIs). To account for multiple testing, P values from the between-group comparisons and longitudinal models were adjusted using the Benjamini–Hochberg false discovery rate (FDR) method. A two-sided *P* value < 0.05 was considered statistically significant.

## 3 Results

### 3.1 Baseline characteristics

The final analysis included 110 eyes from 110 patients who underwent phacoemulsification with monofocal IOL implantation. The mean age of the study population was 65.70 ± 8.12 years, and 56 patients (50.91%) were women. The overall baseline characteristics are summarized in **Table 1**.

**Table 1:** Preoperative characteristics of patients.

Variable	Category	Overall(N = 110)
Sex		
	Male, n (%)	54 (49.09%)
	Female, n (%)	56 (50.91%)
Age (years, mean ± SD)		65.70 ± 8.12
Body mass index (BMI, kg/m^2^)		24.40 ± 2.43
Marital status, n (%)		
	Unmarried/divorced/widowed	14 (12.73%)
	Married	96 (87.27%)
Diabetes mellitus, n (%)		
	No	89 (80.91%)
	Yes	21 (19.09%)
Hypertension, n (%)		
	No	76 (69.09%)
	Yes	34 (30.91%)
Smoking, n (%)		
	No	75 (68.18%)
	Yes	35 (31.82%)
Operated eye, n (%)		
	Left	55 (50.00%)
	Right	55 (50.00%)
Preoperative BCVA (logMAR, median [IQR])		0.5 (0.3, 0.7)
Axial length (mm, mean ± SD)		23.73 ± 1.10
Corneal astigmatism (D, median [IQR])		0.50 (0.25, 1.00)
Corneal endothelial cell density (cells/mm², mean ± SD)		2681.77 ± 249.37
IOL power implanted (D, mean ± SD)		19.65 ± 2.41
TC (mmol/L, mean ± SD)		5.01 ± 1.01
TG (mmol/L, median [IQR])		1.71 (1.22, 2.22)
HDL-C (mmol/L, mean ± SD)		0.90 ± 0.29
LDL-C (mmol/L, mean ± SD)		2.91 ± 0.84

SD, Standard deviation; IQR, interquartile range; BCVA, Best corrected visual acuity; logMAR, Log of the minimum angle of resolution; IOL, Intraocular lens; D, Diopters; TC, total cholesterol; TG, triglyceride; HDL-C, high-density lipoprotein cholesterol; LDL-C, low-density lipoprotein cholesterol.

For baseline comparisons, patients were grouped according to the study-defined lipid classification criteria. TC, TG, and HDL-C were categorized by guideline-based cutoffs, and LDL-C was categorized by risk-adjusted target levels. Overall, baseline characteristics were generally comparable between the TC, TG, and LDL-C groups, whereas some imbalance was observed in the HDL-C grouping. Specifically, there were no significant differences in age, sex, body mass index (BMI), marital status, diabetes, hypertension, smoking, operated eye, preoperative BCVA, axial length (AL), corneal astigmatism, corneal endothelial cell density (CEC), or implanted IOL power (all P > 0.05). In contrast, the HDL-C groups differed in BMI, marital status, and implanted IOL power (all P < 0.05). Baseline comparisons for each lipid grouping are presented in **Supplementary Tables S1**–**S4**.

### 3.2 Postoperative changes in visual quality over time

No intraoperative or postoperative complications were documented among the included eyes during the 1-month follow-up. Over the first postoperative month, both visual acuity and objective visual quality improved. In the adjusted linear mixed-effects models, BCVA improved significantly at postoperative week 1 and month 1 compared with postoperative day 1, with β = -0.044 (95% CI, -0.062 to -0.026; P < 0.001) and β = -0.051 (95% CI, -0.069 to -0.033; P < 0.001), respectively.

This improvement was accompanied by favorable changes in several objective visual quality indices. DLI was higher at postoperative week 1 and month 1 than at postoperative day 1, with β = 1.151 (95% CI, 0.772 to 1.530; P < 0.001) at week 1 and β = 2.324 (95% CI, 1.945 to 2.703; P < 0.001) at month 1. MTF and SR also increased across the total-eye, internal, and corneal measurements. For example, MTF total eye average height increased by 0.042 (95% CI, 0.023 to 0.062; P < 0.001) at week 1 and by 0.084 (95% CI, 0.065 to 0.103; P < 0.001) at month 1, while SR total eye increased by 0.054 (95% CI, 0.035 to 0.074; P < 0.001) at week 1 and by 0.103 (95% CI, 0.083 to 0.122; P < 0.001) at month 1. The same pattern was seen in the internal and corneal measurements. In contrast, OSI and HO total decreased over time. Among the higher-order aberration subcomponents, trefoil and spherical aberration were lower at week 1 and declined further at month 1, whereas coma and secondary astigmatism became significantly lower only at month 1. Detailed results are shown in **Table 2**.

**Table 2:** Overall postoperative temporal changes in visual quality outcomes in linear mixed-effects models.

Outcome	POW1 vs POD1, β (95% CI)	FDR-adjusted P value	POM1 vs POD1, β (95% CI)	FDR-adjusted P value
BCVA	-0.044 (-0.062 to -0.026)	<0.001	-0.051 (-0.069 to -0.033)	<0.001
DLI	1.151 (0.772 to 1.530)	<0.001	2.324 (1.945 to 2.703)	<0.001
MTF cornea average height	0.043 (0.015 to 0.070)	0.003	0.077 (0.049 to 0.104)	<0.001
MTF internal average height	0.045 (0.022 to 0.067)	<0.001	0.077 (0.055 to 0.099)	<0.001
MTF total eye average height	0.042 (0.023 to 0.062)	<0.001	0.084 (0.065 to 0.103)	<0.001
SR cornea	0.058 (0.026 to 0.090)	<0.001	0.105 (0.073 to 0.137)	<0.001
SR internal	0.058 (0.030 to 0.085)	<0.001	0.096 (0.069 to 0.124)	<0.001
SR total eye	0.054 (0.035 to 0.074)	<0.001	0.103 (0.083 to 0.122)	<0.001
OSI	-1.111 (-1.373 to -0.849)	<0.001	-1.483 (-1.745 to -1.221)	<0.001
HO total	-0.101 (-0.176 to -0.025)	0.010	-0.257 (-0.332 to -0.182)	<0.001
Secondary astigmatism	-0.000 (-0.022 to 0.022)	0.986	-0.050 (-0.071 to -0.028)	<0.001
Spherical	-0.017 (-0.033 to -0.002)	0.027	-0.032 (-0.048 to -0.017)	<0.001
Trefoil	-0.079 (-0.120 to -0.038)	<0.001	-0.165 (-0.207 to -0.124)	<0.001
Coma	-0.015 (-0.041 to 0.011)	0.269	-0.055 (-0.081 to -0.029)	<0.001

Data are presented as regression coefficients (β) with 95% confidence intervals (CIs).

Postoperative day 1 (POD1) was treated as the reference time point.

The coefficients for postoperative week 1 (POW1) and postoperative month 1 (POM1) represent the adjusted changes in each outcome relative to POD1.

Models were adjusted for age, body mass index, preoperative BCVA, axial length, corneal astigmatism, sex, diabetes, hypertension, and smoking.

P values were adjusted for multiple comparisons using the Benjamini-Hochberg false discovery rate method.

### 3.3 Between-group differences in postoperative visual quality according to preoperative lipid stratification

Postoperative visual quality was compared between the corresponding TC, TG, HDL-C, and LDL-C groups. The results for LDL-C groups at postoperative month 1 are presented below, and the remaining between-group comparisons are shown in **Supplementary Tables S5**–**S8**. In the covariate-unadjusted comparison at postoperative month 1, the above-target LDL-C group showed less favorable objective optical quality than the at-target group, while no significant difference was observed in BCVA (**Table 3**). Compared with the at-target LDL-C group, the above-target LDL-C group had a higher OSI (1.46 ± 0.46 vs. 1.29 ± 0.37, P = 0.039) and a higher HO total value (P = 0.005). MTF parameters also differed between groups. MTF total eye average height, MTF internal average height, and MTF cornea average height were all lower in the above-target LDL-C group (P = 0.013, P = 0.001, and P = 0.003). Within the internal component, MTF values at 5, 10, 15, 20, 25, and 30 cycles/degree were all lower in patients with higher LDL-C levels (all P < 0.05, **Supplementary Table S8**). SR total eye and SR internal were lower in the above-target LDL-C group (P = 0.009 and P = 0.002).

**Table 3:** Covariate-unadjusted comparison of postoperative month 1 visual outcomes between LDL-C target groups.

Variable	1 month postoperatively		
	At-target LDL-C group (N=60)	Above-target LDL-C group (N=50)	FDR-adjusted P value
DLI	7.79 ± 1.65	7.23 ± 1.92	0.099
OSI	1.29 ± 0.37	1.46 ± 0.46	0.039
BCVA	0.00 (0.00, 0.00)	0.00 (0.00, 0.00)	0.637
HO Total	0.16 ± 0.05	0.21 (0.12, 0.37)	0.005
Coma	0.08 (0.06, 0.11)	0.08 (0.06, 0.10)	0.468
Spherical	0.02 (0.01, 0.04)	0.02 (0.01, 0.03)	0.216
Secondary Astigmatism	0.02 (0.02, 0.03)	0.03 (0.02, 0.04)	0.059
Trefoil	0.09 (0.07, 0.14)	0.11 (0.08, 0.14)	0.202
MTF total eye average height	0.42 ± 0.09	0.38 ± 0.07	0.013
MTF internal average height	0.44 ± 0.11	0.38 ± 0.11	0.001
MTF cornea average height	0.56 ± 0.07	0.51 ± 0.11	0.003
SR total eye	0.26 ± 0.09	0.22 ± 0.07	0.009
SR internal	0.30 ± 0.10	0.25 ± 0.06	0.002
SR cornea	0.41 ± 0.09	0.39 ± 0.08	0.195

Values are presented as mean ± standard deviation or median (interquartile range).

Between-group comparisons were performed using the independent-samples t test or Mann–Whitney U test, as appropriate.

P values were adjusted for multiple comparisons using the Benjamini–Hochberg false discovery rate method. An FDR-adjusted P value < 0.05 was considered statistically significant.

BCVA, best-corrected visual acuity; DLI, dysfunctional lens index; OSI, objective scatter index; HO, higher-order aberration; MTF, modulation transfer function; LDL-C, low-density lipoprotein cholesterol.

### 3.4 Multivariable-adjusted sensitivity analysis of postoperative month 1 LDL-C group differences

To assess whether the postoperative month 1 differences between LDL-C target groups were robust to adjustment for potential confounders, multivariable linear regression analyses were performed using the same covariates as in the longitudinal models. As shown in **Table 4**, above-target LDL-C remained associated with higher HO Total (β = 0.121, 95% CI, 0.034 to 0.207; FDR-adjusted P = 0.049) and lower SR Internal (β = -0.050, 95% CI, -0.086 to -0.014; FDR-adjusted P = 0.049). MTF Internal Avg Height and MTF Corneal Avg Height were also lower in the above-target group, but these associations did not remain statistically significant after FDR correction (FDR-adjusted P = 0.071 for both). The other major optical quality outcomes were not significant after FDR adjustment.

**Table 4:** Multivariable-adjusted differences in postoperative month 1 visual outcomes between LDL-C target groups.

Outcome	Adjusted β	HC3 95% CI	Raw P value	FDR P value
BCVA	0.013	-0.017 to 0.043	0.381	0.489
HO Total	0.121	0.034 to 0.207	0.007	0.049
Coma	-0.015	-0.058 to 0.027	0.480	0.560
Spherical	-0.011	-0.028 to 0.006	0.184	0.322
Secondary Astigmatism	-0.004	-0.020 to 0.012	0.636	0.636
Trefoil	0.017	-0.041 to 0.076	0.562	0.605
DLI	-0.419	-1.302 to 0.463	0.348	0.489
OSI	0.161	-0.050 to 0.373	0.134	0.267
MTF Total Eye Avg Height	-0.040	-0.081 to 0.002	0.060	0.168
MTF Internal Avg Height	-0.066	-0.119 to -0.012	0.016	0.071
MTF Corneal Avg Height	-0.053	-0.097 to -0.008	0.020	0.071
SR Total Eye	-0.036	-0.077 to 0.006	0.089	0.208
SR Internal	-0.050	-0.086 to -0.014	0.007	0.049
SR Cornea	-0.020	-0.065 to 0.025	0.384	0.489

Adjusted β represents the estimated difference in each postoperative month 1 outcome for the above-target LDL-C group relative to the at-target LDL-C group, which was used as the reference. Multivariable linear regression models were adjusted for age, sex, BMI, smoking status, diabetes, hypertension, preoperative BCVA, axial length, and corneal astigmatism. HC3 heteroscedasticity-robust standard errors were used to calculate 95% confidence intervals and raw P values. P values were further adjusted for multiple comparisons across the 14 summary outcomes using the Benjamini–Hochberg false discovery rate method. An FDR-adjusted P value < 0.05 was considered statistically significant.

LDL-C, low-density lipoprotein cholesterol; BCVA, best-corrected visual acuity; HO, higher-order aberration; DLI, dysfunctional lens index; OSI, objective scatter index; MTF, modulation transfer function; SR, Strehl ratio; CI, confidence interval; FDR, false discovery rate.

### 3.5 Correlations between preoperative lipid parameters and postoperative month 1 visual quality indices

Spearman correlation analysis at postoperative month 1 is shown in **Figure 2** and **Supplementary Table S9**. TC, TG, and LDL-C were generally associated with less favorable optical quality. These correlations were mainly reflected in lower MTF and SR values. No significant association was found between HDL-C and any visual quality index. Representative findings included negative correlations of TG with MTF internal average height (r = -0.31, P = 0.001) and SR internal (r = -0.33, P < 0.001). LDL-C was negatively correlated with MTF total eye average height (r = -0.24, P = 0.012) and SR internal (r = -0.24, P = 0.011). It was positively correlated with HO total (r = 0.24, P = 0.010). No significant associations were observed between any lipid parameter and DLI or OSI.

**Figure 2:**
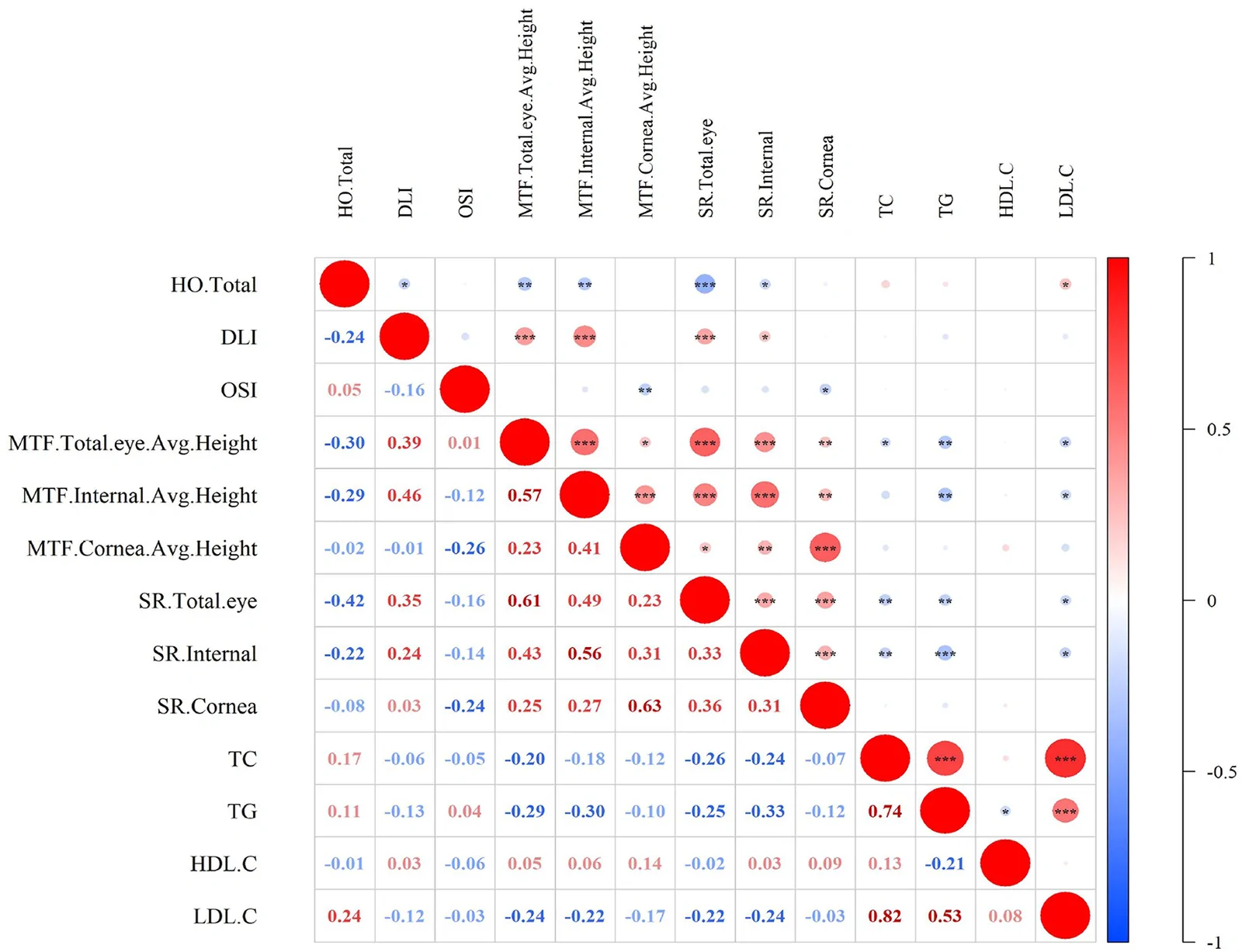
Heatmap of Spearman correlations between preoperative lipid parameters and postoperative month-1 visual quality indices. Spearman correlation coefficients are shown for the associations between preoperative lipid parameters and postoperative month-1 visual quality indices. Red indicates positive correlations and blue indicates negative correlations, with color intensity and circle size reflecting the strength of the correlation. *P < 0.05, **P < 0.01, ***P < 0.001. TC, total cholesterol; TG, triglycerides; HDL-C, high-density lipoprotein cholesterol; LDL-C, low-density lipoprotein cholesterol; DLI, dysfunctional lens index; OSI, objective scatter index; HO, higher-order aberration; MTF, modulation transfer function; SR, Strehl ratio.

In addition, correlations were also observed among the lipid parameters themselves. The strongest intercorrelations were found between TC and LDL-C (r = 0.821, P < 0.001), TC and TG (r = 0.741, P < 0.001), and TG and LDL-C (r = 0.534, P < 0.001).

### 3.6 Longitudinal associations of LDL-C with postoperative visual outcomes

In the adjusted linear mixed-effects models, LDL-C was associated with postoperative BCVA, while a significant interaction effect was observed for spherical aberration at month 1 (**Table 5**). For BCVA, the association with LDL-C appeared to be time-dependent. A significant inverse association with LDL-C was observed at postoperative day 1 (β = -0.046, 95% CI, -0.069 to -0.023, P = 0.002), but this association was attenuated at postoperative week 1 and month 1. Accordingly, significant interaction effects were observed at both week 1 and month 1, with β = 0.042 (95% CI, 0.021 to 0.062, P = 0.003) at week 1 and β = 0.033 (95% CI, 0.012 to 0.054, P = 0.017) at month 1.

**Table 5:** Longitudinal associations of LDL-C with postoperative BCVA and spherical aberration in linear mixed-effects models.

Outcome	POD1 slope (95% CI)	FDR-adjusted P value	POW1 slope, β (95% CI)	P value	POM1 slope, β (95% CI)	FDR-adjusted P value
BCVA	-0.046 (-0.069 to -0.023)	0.002	-0.005 (-0.028 to 0.018)	0.765	-0.013 (-0.036 to 0.010)	0.919
Spherical aberration	0.017 (0.003 to 0.032)	0.135	-0.007 (-0.022 to 0.007)	0.765	-0.013 (-0.028 to 0.001)	0.919
			POW1 vs POD1 interaction, β (95% CI)	P value	POM1 vs POD1 interaction, β (95% CI)	P value
BCVA			0.042 (0.021 to 0.062)	0.003	0.033 (0.012 to 0.054)	0.017
Spherical aberration			-0.024 (-0.042 to -0.007)	0.050	-0.030 (-0.048 to -0.013)	0.012

Data are presented as regression coefficients (β) with 95% confidence intervals (CIs).

Postoperative day 1 (POD1) was treated as the reference time point.

The POD1, POW1, and POM1 slopes represent the associations between LDL-C and the outcomes at POD1, POW1, and POM1, respectively.

Interaction terms indicate the change in the LDL-C slope at POW1 or POM1 relative to POD1.

Models were adjusted for age, body mass index, preoperative BCVA, axial length, corneal astigmatism, sex, diabetes, hypertension, and smoking.

P values were adjusted for multiple comparisons using the Benjamini-Hochberg false discovery rate method.

For spherical aberration, no significant association with LDL-C was observed at postoperative day 1, week 1, or month 1. However, the interaction term was significant at month 1 (β = -0.030, 95% CI, -0.048 to -0.013, P = 0.012), whereas the week 1 interaction did not reach statistical significance (P = 0.050).

No significant longitudinal associations were observed for TC, TG, or HDL-C after FDR correction. The full longitudinal results for all four lipid parameters are shown in **Supplementary Table S10**. Because the HDL-C groups differed in marital status and IOL power at baseline, both variables were additionally included in the HDL-C longitudinal models. This additional adjustment did not materially alter the HDL-C-related estimates, and no association remained statistically significant after FDR correction (**Supplementary Table S11**).

## 4 Discussion

The present study examined the association between preoperative lipid status and early postoperative visual quality after cataract surgery. To facilitate interpretation of the optical parameters, MTF reflects how well image contrast is transferred through the optical system (17). SR indicates how closely retinal image quality approaches the diffraction-limited ideal (17). Higher MTF and SR values indicate better optical quality (17). OSI quantifies intraocular light scatter, while HOA reflects wavefront distortion (18, 19). Higher OSI and HOA values indicate poorer optical quality (18, 19). DLI is an iTrace-derived index of internal optical quality, with higher values indicating better optical performance (20). In this study, visual acuity and objective optical quality improved progressively during the first month after cataract surgery. Among the four lipid parameters, LDL-C showed the clearest pattern of association with early postoperative objective optical quality, particularly at postoperative month 1. At that time point, the above-target LDL-C group had higher OSI and HO total values and lower MTF and SR values than the at-target group, whereas BCVA did not differ significantly between groups. After adjustment for potential confounders, the associations with higher HO Total and lower SR Internal remained significant, whereas the other postoperative month 1 differences were attenuated. TC and TG also showed some associations with postoperative optical quality, but the evidence was less consistent than that for LDL-C. Correlation analysis further showed that TC, TG, and LDL-C were associated with less favorable optical quality, while no clear association was observed for HDL-C. In contrast, fewer significant associations remained in the adjusted longitudinal models. This suggests that the postoperative month 1 group differences did not completely parallel the longitudinal findings.

The postoperative month 1 findings are noteworthy because differences in objective optical quality were observed despite similar BCVA between LDL-C groups. Although several optical parameters differed in the unadjusted comparison, only HO Total and SR Internal remained significantly associated with LDL-C group after multivariable adjustment and FDR correction. This pattern suggests that the association with LDL-C may be more apparent in objective optical quality measures than in BCVA at postoperative month 1. Previous studies have similarly shown that postoperative visual recovery cannot be fully described by acuity alone and that objective optical quality measures provide additional information on image quality after cataract surgery (21, 22).

In the longitudinal models, fewer LDL-C-related associations remained significant after covariate adjustment and FDR correction. LDL-C was associated mainly with BCVA, while only a limited time-dependent association was observed for spherical aberration. The postoperative month 1 analyses and the longitudinal models addressed different aspects of the data and should therefore be interpreted separately. The between-group comparison at postoperative month 1 focused on a single postoperative time point. This analysis was intended to identify group differences at a clinically relevant stage of recovery. The longitudinal models addressed a different question, whether LDL-C was independently associated with recovery trajectories across the entire follow-up period. Differences in model structure, repeated-measure variability, and the use of categorical versus continuous LDL-C measures may also have contributed to the different patterns observed across analyses (23–25). Overall, the stronger postoperative month 1 signal and the more limited longitudinal findings suggest that the observed LDL-C association should be regarded as an early signal requiring prospective confirmation rather than as evidence of a stable longitudinal effect. LDL-C was therefore emphasized in the present study because it showed the clearest pattern at postoperative month 1. It is also the primary lipid target in current clinical guidelines, which gives the target-based grouping direct clinical relevance.

Several factors may contribute to the observed association between LDL-C and postoperative optical quality. One possible explanation involves the ocular surface. Previous studies suggest that dyslipidemia is associated with dry eye disease and meibomian gland dysfunction (26–28). One proposed explanation is that abnormal lipid metabolism may influence meibum composition and meibomian gland function, although this link has not been fully established (29–31). Reduced meibum secretion or altered meibum composition can disrupt the tear-film lipid layer, increase tear evaporation, and reduce tear-film stability (32). Because the tear film forms the first refractive surface of the eye, instability or break-up can make the anterior optical surface irregular and increase higher-order aberrations and light scatter (33). Tear-film instability may also increase variability in corneal astigmatism measurements, which can further affect the assessment of postoperative optical quality (34). Cataract surgery itself can transiently disturb the ocular surface and meibomian gland function (35–37). Systemic inflammation may represent another possible pathway. Dyslipidemia has been associated with chronic low-grade inflammation (9). Experimental studies and reviews suggest that cholesterol accumulation may activate inflammatory signaling pathways (38–40). Elevated LDL-C may therefore reflect a broader metabolic and inflammatory background that could influence the postoperative ocular environment and early optical recovery. Together, these ocular-surface and inflammatory pathways provide a possible link between systemic lipid status and early postoperative optical quality.

In clinical practice, LDL-C has one practical advantage: it is already available from routine preoperative blood tests. In the present study, patients with above-target LDL-C tended to show less favorable early optical recovery. This finding does not support using LDL-C alone to predict postoperative visual quality. Consistent with current perioperative cardiovascular guidelines, elevated LDL-C should be managed according to the patient’s overall cardiovascular risk and established treatment indications, and should not by itself prompt postponement of cataract surgery (41). Even so, it may help clinicians pay closer attention to early optical recovery, especially in patients with high expectations for postoperative visual quality.

A strength of this study is the use of objective optical quality measures alongside BCVA, which allowed postoperative differences not captured by visual acuity alone to be identified. The group-based, correlation, and longitudinal analyses provided complementary views of the association between lipid status and postoperative optical outcomes.

Taken together, these findings suggest a possible link between preoperative lipid status and early postoperative optical quality after cataract surgery. The association observed with LDL-C further suggests that systemic metabolic factors may contribute, alongside ocular and surgical factors, to individual differences in early optical recovery.

## 5 Limitations

This study has several limitations. First, it was a retrospective, single-center study with a relatively small sample of 110 eyes, and all surgeries were performed by the same surgeon. No *a priori* sample size calculation was performed because all eligible patients during the study period were included. These factors limit the generalizability of the findings, may have reduced the ability to detect modest associations, and preclude causal inference. Second, follow-up ended at one month after surgery, so the relationship between lipid status and longer-term optical outcomes could not be assessed. Third, detailed dry-eye and ocular-surface assessments, serial postoperative corneal astigmatism measurements, and inflammatory biomarkers were not systematically available. Although baseline corneal astigmatism was included in the adjusted models, the possible contribution of postoperative tear-film instability to optical variability could not be directly evaluated. The ocular-surface and inflammatory pathways discussed above therefore remain possible explanations rather than mechanisms demonstrated by this study. Fourth, the adjusted models included major demographic, systemic, and ocular covariates, and additional sensitivity analyses were performed for relevant baseline imbalances. Nevertheless, residual or unmeasured confounding cannot be excluded. In addition, the study was based on routine serum lipid measurements rather than a comprehensive assessment of metabolic function, so the broader metabolic mechanisms underlying the observed associations could not be determined. Finally, all patients received the same monofocal IOL model. This reduced variability related to IOL design but may limit the generalizability of the findings to other IOL types and clinical settings.

## 6 Conclusion

In this cohort, preoperative lipid status was associated with early postoperative objective optical quality after cataract surgery. Among the routine lipid parameters, the clearest signal was observed for LDL-C, particularly at postoperative month 1. However, the associations were more limited in the adjusted longitudinal analyses. These findings suggest a possible relationship between preoperative lipid status and early optical recovery and should be considered hypothesis-generating. Larger prospective studies are needed to confirm these associations and clarify the underlying mechanisms.

## Data Availability

The raw data supporting the conclusions of this article will be made available by the authors, without undue reservation.
