# Supplementary material for: Association between preoperative lipid profile and early postoperative objective optical quality after cataract surgery
Source: Front Endocrinol (Lausanne). 2026 Oct 9;17:1887440. doi: 10.3389/fendo.2026.1887440 (PMC13690559; doi:10.3389/fendo.2026.1887440)
Supplement: Supplementary file 1 [file Table1.docx]

**Supplementary Materials**

| **Supplementary Table S1. Baseline characteristics according to TC categories** | | | | |
| --- | --- | --- | --- | --- |
| Variable | Overall（N=110） | Low TC group (N=60) | High TC group (N=50) | P value |
| Sex |  |  |  | 0.347 |
| Male, n (n%) | 54 (49.09%) | 27 (45.00%) | 27 (54.00%) |  |
| Female, n (n%) | 56 (50.91%) | 33 (55.00%) | 23 (46.00%) |  |
| Age (years, mean ± SD) | 65.70 ± 8.12 | 66.80 ± 7.83 | 64.38 ± 8.15 | 0.120 |
| Body mass index (BMI, kg/m^2^) | 24.40 ± 2.43 | 24.23 ± 2.56 | 24.60 ± 2.27 | 0.435 |
| Marital status, n (%) |  |  |  | 0.347 |
| Unmarried / divorced / widowed | 14 (12.73%) | 6 (10.00%) | 8 (16.00%) |  |
| Married | 96 (87.27%) | 54 (90.00%) | 42 (84.00%) |  |
| Diabetes mellitus, n (%) |  |  |  | 0.215 |
| No | 89 (80.91%) | 46 (76.67%) | 43 (86.00%) |  |
| Yes | 21 (19.09%) | 14 (23.33%) | 7 (14.00%) |  |
| Hypertension, n (%) |  |  |  | 0.309 |
| No | 76 (69.09%) | 39 (65.00%) | 37 (74.00%) |  |
| Yes | 34 (30.91%) | 21 (35.00%) | 13 (26.00%) |  |
| Smoking, n (%) |  |  |  | 0.390 |
| No | 75 (68.18%) | 43 (71.67%) | 32 (64.00%) |  |
| Yes | 35 (31.82%) | 17 (28.33%) | 18 (36.00%) |  |
| Operated eye, n (%) |  |  |  | 0.702 |
| Left | 55 (50.00%) | 31 (51.67%) | 24 (48.00%) |  |
| Right | 55 (50.00%) | 29 (48.33%) | 26 (52.00%) |  |
| Preoperative BCVA (logMAR, median [IQR]) | 0.5 (0.3, 0.7) | 0.40 (0.30, 0.70) | 0.60 (0.33, 0.70) | 0.125 |
| Axial length (mm, mean ± SD) | 23.73 ± 1.10 | 23.74 ± 1.00 | 23.72 ± 1.23 | 0.938 |
| Corneal astigmatism (D, median [IQR]) | 0.50 (0.25, 1.00) | 0.50(0.25, 1.00) | 0.63 (0.25, 1.19) | 0.740 |
| Corneal endothelial cell density (cells/mm², mean ± SD) | 2681.77 ± 249.37 | 2707.57 ± 204.32 | 2650.82 ± 293.72 | 0.236 |
| IOL power implanted (D, mean ± SD) | 19.65 ± 2.41 | 19.30 ± 2.55 | 20.07 ± 2.17 | 0.094 |
| TC (mmol/L, mean ± SD) | 5.01 ± 1.01 | 4.30 ± 0.67 | 5.86 ± 0.63 | <0.001 |
| SD: Standard deviation; IQR: interquartile range; BCVA: Best corrected visual acuity; logMAR: Log of the minimum angle of resolution; IOL: Intraocular lens; D: Diopters; TC, total cholesterol. | | | | |
|  |  |  |  |  |

| **Supplementary Table S2. Baseline characteristics according to TG categories** | | | | |
| --- | --- | --- | --- | --- |
| Variable | Overall（N=110） | Low TG group (N=55) | High TG group (N=55) | P value |
| Sex |  |  |  | 0.127 |
| Male, n (n%) | 54 (49.09%) | 23 (41.82%) | 31 (56.36%) |  |
| Female, n (n%) | 56 (50.91%) | 32 (58.18%) | 24 (43.64%) |  |
| Age (years, mean ± SD) | 65.70 ± 8.12 | 66.65 ± 8.09 | 64.75 ± 8.12 | 0.222 |
| Body mass index (BMI, kg/m^2^) | 24.40 ± 2.43 | 24.15 ± 2.59 | 24.66 ± 2.28 | 0.276 |
| Marital status, n (%) |  |  |  | 0.567 |
| Unmarried / divorced / widowed | 14 (12.73%) | 6 (10.91%) | 8 (14.55%) |  |
| Married | 96 (87.27%) | 49 (89.09%) | 47 (85.45%) |  |
| Diabetes mellitus, n (%) |  |  |  | 0.808 |
| No | 89 (80.91%) | 44 (80.00%) | 45 (81.82%) |  |
| Yes | 21 (19.09%) | 11 (20.00%) | 10 (18.18%) |  |
| Hypertension, n (%) |  |  |  | 0.680 |
| No | 76 (69.09%) | 37 (67.27%) | 39 (70.91%) |  |
| Yes | 34 (30.91%) | 18 (32.73%) | 16 (29.09%) |  |
| Smoking, n (%) |  |  |  | 0.306 |
| No | 75 (68.18%) | 40 (72.73%) | 35 (63.64%) |  |
| Yes | 35 (31.82%) | 15 (27.27%) | 20 (36.36%) |  |
| Operated eye, n (%) |  |  |  | 0.849 |
| Left | 55 (50.00%) | 28 (50.91%) | 27 (49.09%) |  |
| Right | 55 (50.00%) | 27 (49.09%) | 28 (50.91%) |  |
| Preoperative BCVA (logMAR, median [IQR]) | 0.5 (0.3, 0.7) | 0.40 (0.35, 0.70) | 0.50 (0.30, 0.70) | 0.239 |
| Axial length (mm, mean ± SD) | 23.73 ± 1.10 | 23.71 ± 1.09 | 23.70 ± 1.13 | 0.847 |
| Corneal astigmatism (D, median [IQR]) | 0.50 (0.25, 1.00) | 0.50 (0.25, 1.00) | 0.75 (0.25, 1.00) | 0.760 |
| Corneal endothelial cell density (cells/mm², mean ± SD) | 2681.77 ± 249.37 | 2706.53 ± 213.48 | 2657.02 ± 280.53 | 0.300 |
| IOL power implanted (D, mean ± SD) | 19.65 ± 2.41 | 19.48 ± 2.72 | 19.82 ± 2.06 | 0.464 |
| TG (mmol/L, median [IQR]) | 1.71 (1.22, 2.22) | 1.21 (0.82, 1.45) | 2.22 (1.95, 3.15) | <0.001 |
| SD: Standard deviation; IQR: interquartile range; BCVA: Best corrected visual acuity; logMAR: Log of the minimum angle of resolution; IOL: Intraocular lens; D: Diopters; TG, triglyceride. | | | | |
|  |  |  |  |  |

| **Supplementary Table S3. Baseline characteristics according to HDL-C categories** | | | | |
| --- | --- | --- | --- | --- |
| Variable | Overall（N=110） | Low HDL-C group (N=88) | Non-low HDL-C group (N=22) | P value |
| Sex |  |  |  | 0.182 |
| Male, n (n%) | 54 (49.09%) | 46 (52.27%) | 8 (36.36%) |  |
| Female, n (n%) | 56 (50.91%) | 42 (47.73%) | 14 (63.64%) |  |
| Age (years, mean ± SD) | 65.70 ± 8.12 | 65.58 ± 8.24 | 66.18 ± 7.82 | 0.757 |
| Body mass index (BMI, kg/m^2^) | 24.40 ± 2.43 | 24.71 ± 2.47 | 23.16 ± 1.81 | 0.002 |
| Marital status, n (%) |  |  |  | 0.033 |
| Unmarried / divorced / widowed | 14 (12.73%) | 8 (9.09%) | 6 (27.27%) |  |
| Married | 96 (87.27%) | 80 (90.91%) | 16 (72.73%) |  |
| Diabetes mellitus, n (%) |  |  |  | 1.000 |
| No | 89 (80.91%) | 71 (80.68%) | 18 (81.82%) |  |
| Yes | 21 (19.09%) | 17 (19.32%) | 4 (18.18%) |  |
| Hypertension, n (%) |  |  |  | 0.680 |
| No | 76 (69.09%) | 60 (68.18%) | 16 (72.73%) |  |
| Yes | 34 (30.91%) | 28 (31.82%) | 6 (27.27%) |  |
| Smoking, n (%) |  |  |  | 0.125 |
| No | 75 (68.18%) | 63 (71.59%) | 12 (54.55%) |  |
| Yes | 35 (31.82%) | 25 (28.41%) | 10 (45.45%) |  |
| Operated eye, n (%) |  |  |  | 0.340 |
| Left | 55 (50.00%) | 42 (47.73%) | 13 (59.09%) |  |
| Right | 55 (50.00%) | 46 (52.27%) | 9 (40.91%) |  |
| Preoperative BCVA (logMAR, median [IQR]) | 0.5 (0.3, 0.7) | 0.50 (0.30, 0.70) | 0.50 (0.33, 0.70) | 0.669 |
| Axial length (mm, mean ± SD) | 23.73 ± 1.10 | 23.78 ± 1.02 | 23.52 ± 1.41 | 0.424 |
| Corneal astigmatism (D, median [IQR]) | 0.50 (0.25, 1.00) | 0.50 (0.25, 1.00) | 0.75 (0.50, 1.00) | 0.414 |
| Corneal endothelial cell density (cells/mm², mean ± SD) | 2681.77 ± 249.37 | 2688.39 ± 235.34 | 2655.32 ± 303.95 | 0.580 |
| IOL power implanted (D, mean ± SD) | 19.65 ± 2.41 | 19.33 ± 2.35 | 20.91 ± 2.25 | 0.005 |
| HDL-C (mmol/L, mean ± SD) | 0.90 ± 0.29 | 0.79 ± 0.12 | 1.34 ± 0.33 | <0.001 |
| SD: Standard deviation; IQR: interquartile range; BCVA: Best corrected visual acuity; logMAR: Log of the minimum angle of resolution; IOL: Intraocular lens; D: Diopters; HDL-C, high-density lipoprotein cholesterol. | | | | |
|  |  |  |  |  |

| **Supplementary Table S4. Baseline characteristics according to LDL-C categories** | | | | |
| --- | --- | --- | --- | --- |
| Variable | Overall（N=110） | at-target LDL-C group (N=60) | above-target LDL-C group (N=50) | P value |
| Sex |  |  |  | 0.347 |
| Male, n (n%) | 54 (49.09%) | 27 (45.00%) | 27 (54.00%) |  |
| Female, n (n%) | 56 (50.91%) | 33 (55.00%) | 23 (46.00%) |  |
| Age (years, mean ± SD) | 65.70 ± 8.12 | 65.83 ± 8.49 | 65.54 ± 7.74 | 0.851 |
| Body mass index (BMI, kg/m^2^) | 24.40 ± 2.43 | 24.15 ± 2.53 | 24.70 ± 2.29 | 0.24 |
| Marital status, n (%) |  |  |  | 0.347 |
| Unmarried / divorced / widowed | 14 (12.73%) | 6 (10.00%) | 8 (16.00%) |  |
| Married | 96 (87.27%) | 54 (90.00%) | 42 (84.00%) |  |
| Diabetes mellitus, n (%) |  |  |  | 0.790 |
| No | 89 (80.91%) | 48 (80.00%) | 41 (82.00%) |  |
| Yes | 21 (19.09%) | 12 (20.00%) | 9 (18.00%) |  |
| Hypertension, n (%) |  |  |  | 0.522 |
| No | 76 (69.09%) | 43 (71.67%) | 33 (66.00%) |  |
| Yes | 34 (30.91%) | 17 (28.33%) | 17 (34.00%) |  |
| Smoking, n (%) |  |  |  | 0.093 |
| No | 75 (68.18%) | 45 (75.00%) | 30 (60.00%) |  |
| Yes | 35 (31.82%) | 15 (25.00%) | 20 (40.00%) |  |
| Operated eye, n (%) |  |  |  | 0.702 |
| Left | 55 (50.00%) | 29 (48.33%) | 26 (52.00%) |  |
| Right | 55 (50.00%) | 31 (51.67%) | 24 (48.00%) |  |
| Preoperative BCVA (logMAR, median [IQR]) | 0.5 (0.3, 0.7) | 0.40 (0.30, 0.70) | 0.60 (0.33, 0.70) | 0.096 |
| Axial length (mm, mean ± SD) | 23.73 ± 1.10 | 23.72 ± 1.03 | 23.74 ± 1.20 | 0.950 |
| Corneal astigmatism (D, median [IQR]) | 0.50 (0.25, 1.00) | 0.50 (0.25, 1.00) | 0.63 (0.50, 1.00) | 0.500 |
| Corneal endothelial cell density (cells/mm², mean ± SD) | 2681.77 ± 249.37 | 2674.95 ± 207.97 | 2689.96 ± 293.46 | 0.750 |
| IOL power implanted (D, mean ± SD) | 19.65 ± 2.41 | 19.39 ± 2.61 | 19.96 ± 2.12 | 0.220 |
| LDL-C (mmol/L, mean ± SD) | 2.91 ± 0.84 | 2.38 ± 0.65 | 3.54 ± 0.56 | <0.001 |
| SD: Standard deviation; IQR: interquartile range; BCVA: Best corrected visual acuity; logMAR: Log of the minimum angle of resolution; IOL: Intraocular lens; D: Diopters; LDL-C, low-density lipoprotein cholesterol. | | | | |
|  |  |  |  |  |

| **Supplementary Table S5. Comparison of postoperative visual quality parameters between the low and high TC groups** | | | | | | | | | |
| --- | --- | --- | --- | --- | --- | --- | --- | --- | --- |
|  | 1 day postoperatively | |  | 1 week postoperatively | |  | 1 month postoperatively | |  |
| Variable | Low TC group (N=60) | High TC group (N=50) | P value | Low TC group (N=60) | High TC group (N=50) | P value | Low TC group (N=60) | High TC group (N=50) | P value |
| DLI | 5.23 ± 1.90 | 5.19 ± 2.37 | 0.928 | 6.64 ± 2.12 | 6.03 ± 2.46 | 0.166 | 7.78 ± 1.78 | 7.24 ± 1.78 | 0.116 |
| OSI | 2.78 ± 1.63 | 2.94 ± 1.61 | 0.596 | 1.71 ± 0.79 | 1.78 ± 0.84 | 0.681 | 1.32 ± 0.39 | 1.42 ± 0.45 | 0.232 |
| BCVA | 0.00 (0.00, 0.10) | 0.00 (0.00, 0.10) | 0.174 | 0.00 (0.00, 0.00) | 0.00 (0.00, 0.08) | 0.494 | 0.00 (0.00, 0.00) | 0.00 (0.00, 0.00) | 0.637 |
| HO total | 0.31 (0.18, 0.47) | 0.32 (0.20, 0.47) | 0.540 | 0.20 (0.14, 0.27) | 0.22 (0.13, 0.32) | 0.394 | 0.15 (0.12, 0.20) | 0.21 (0.13, 0.36) | 0.011 |
| Coma | 0.11 (0.06, 0.19) | 0.13 (0.07, 0.19) | 0.296 | 0.08 (0.05, 0.13) | 0.12 (0.04, 0.17) | 0.303 | 0.07 (0.06, 0.11) | 0.08 (0.06, 0.10) | 0.966 |
| Spherical | 0.03 (0.01, 0.07) | 0.05 (0.02, 0.08) | 0.221 | 0.02 (0.01, 0.03) | 0.02 (0.01, 0.04) | 0.703 | 0.02 (0.01, 0.04) | 0.02 (0.01, 0.03) | 0.149 |
| Secondary Astigmatism | 0.04 (0.02, 0.07) | 0.05 (0.03, 0.09) | 0.282 | 0.03 (0.02, 0.07) | 0.04 (0.02, 0.11) | 0.425 | 0.02 (0.02, 0.03) | 0.03 (0.02, 0.04) | 0.363 |
| Trefoil | 0.18 (0.11, 0.33) | 0.21 (0.13, 0.38) | 0.296 | 0.13 (0.09, 0.20) | 0.15 (0.07, 0.27) | 0.585 | 0.09 (0.07, 0.13) | 0.11 (0.08, 0.14) | 0.326 |
| MTF total eye |  |  |  |  |  |  |  |  |  |
| average height | 0.31 ± 0.09 | 0.32 ± 0.12 | 0.796 | 0.36 ± 0.11 | 0.35 ± 0.11 | 0.446 | 0.42 ± 0.09 | 0.37 ± 0.06 | 0.005 |
| 5 cycles/degree | 0.58 ± 0.17 | 0.56 ± 0.21 | 0.75 | 0.64 ± 0.18 | 0.61 ± 0.19 | 0.38 | 0.71 ± 0.13 | 0.68 ± 0.11 | 0.137 |
| 10 cycles/degree | 0.30 ± 0.14 | 0.31 ± 0.18 | 0.679 | 0.38 ± 0.16 | 0.37 ± 0.16 | 0.732 | 0.46 ± 0.13 | 0.41 ± 0.10 | 0.025 |
| 15 cycles/degree | 0.19 ± 0.10 | 0.20 ± 0.13 | 0.397 | 0.25 ± 0.13 | 0.25 ± 0.14 | 0.961 | 0.31 ± 0.12 | 0.26 ± 0.10 | 0.029 |
| 20 cycles/degree | 0.13 ± 0.07 | 0.14 ± 0.08 | 0.751 | 0.18 ± 0.10 | 0.18 ± 0.11 | 0.987 | 0.23 ± 0.09 | 0.20 ± 0.09 | 0.065 |
| 25 cycles/degree | 0.11 ± 0.05 | 0.11 ± 0.06 | 0.824 | 0.15 ± 0.08 | 0.15 ± 0.09 | 0.983 | 0.18 ± 0.08 | 0.16 ± 0.07 | 0.143 |
| 30 cycles/degree | 0.09 ± 0.04 | 0.09 ± 0.05 | 0.853 | 0.12 ± 0.07 | 0.12 ± 0.07 | 0.94 | 0.15 ± 0.06 | 0.13 ± 0.06 | 0.112 |
| MTF internal |  |  |  |  |  |  |  |  |  |
| average height | 0.34 ± 0.12 | 0.34 ± 0.15 | 0.903 | 0.39 ± 0.12 | 0.37 ± 0.14 | 0.322 | 0.44 ± 0.11 | 0.38 ± 0.10 | 0.007 |
| 5 cycles/degree | 0.60 ± 0.21 | 0.58 ± 0.23 | 0.605 | 0.68 ± 0.18 | 0.63 ± 0.22 | 0.188 | 0.71 ± 0.18 | 0.66 ± 0.17 | 0.170 |
| 10 cycles/degree | 0.35 ± 0.18 | 0.34 ± 0.21 | 0.909 | 0.43 ± 0.17 | 0.40 ± 0.20 | 0.346 | 0.47 ± 0.18 | 0.42 ± 0.15 | 0.086 |
| 15 cycles/degree | 0.22 ± 0.14 | 0.23 ± 0.16 | 0.722 | 0.28 ± 0.13 | 0.27 ± 0.16 | 0.488 | 0.33 ± 0.15 | 0.27 ± 0.12 | 0.030 |
| 20 cycles/degree | 0.16 ± 0.11 | 0.18 ± 0.13 | 0.489 | 0.21 ± 0.11 | 0.21 ± 0.19 | 0.932 | 0.25 ± 0.12 | 0.20 ± 0.09 | 0.026 |
| 25 cycles/degree | 0.14 ± 0.10 | 0.14 ± 0.11 | 0.957 | 0.17 ± 0.09 | 0.16 ± 0.10 | 0.925 | 0.20 ± 0.10 | 0.16 ± 0.07 | 0.026 |
| 30 cycles/degree | 0.11 ± 0.07 | 0.12 ± 0.09 | 0.608 | 0.14 ± 0.08 | 0.13 ± 0.09 | 0.438 | 0.16 ± 0.09 | 0.13 ± 0.06 | 0.042 |
| MTF cornea |  |  |  |  |  |  |  |  |  |
| average height | 0.46 ± 0.14 | 0.47 ± 0.12 | 0.833 | 0.49 ± 0.11 | 0.52 ± 0.10 | 0.194 | 0.55 ± 0.08 | 0.53 ± 0.10 | 0.167 |
| 5 cycles/degree | 0.73 ± 0.15 | 0.74 ± 0.15 | 0.827 | 0.78 ± 0.13 | 0.79 ± 0.13 | 0.834 | 0.82 ± 0.08 | 0.80 ± 0.11 | 0.144 |
| 10 cycles/degree | 0.50 ± 0.19 | 0.52 ± 0.16 | 0.507 | 0.56 ± 0.15 | 0.60 ± 0.14 | 0.229 | 0.62 ± 0.13 | 0.60 ± 0.14 | 0.432 |
| 15 cycles/degree | 0.38 ± 0.18 | 0.37 ± 0.14 | 0.915 | 0.43 ± 0.14 | 0.44 ± 0.12 | 0.618 | 0.46 ± 0.13 | 0.46 ± 0.13 | 0.962 |
| 20 cycles/degree | 0.28 ± 0.13 | 0.27 ± 0.10 | 0.679 | 0.33 ± 0.13 | 0.34 ± 0.11 | 0.510 | 0.38 ± 0.10 | 0.38 ± 0.11 | 0.962 |
| 25 cycles/degree | 0.21 ± 0.09 | 0.20 ± 0.07 | 0.722 | 0.26 ± 0.11 | 0.27 ± 0.09 | 0.811 | 0.31 ± 0.10 | 0.30 ± 0.10 | 0.704 |
| 30 cycles/degree | 0.18 ± 0.08 | 0.17 ± 0.06 | 0.532 | 0.22 ± 0.10 | 0.22 ± 0.08 | 0.746 | 0.26 ± 0.08 | 0.25 ± 0.09 | 0.830 |
| SR total eye | 0.14 ± 0.08 | 0.14 ± 0.09 | 0.983 | 0.20 ± 0.12 | 0.19 ± 0.09 | 0.667 | 0.27 ± 0.09 | 0.21 ± 0.06 | <0.001 |
| SR internal | 0.17 ± 0.13 | 0.19 ± 0.16 | 0.696 | 0.24 ± 0.13 | 0.23 ± 0.14 | 0.886 | 0.30 ± 0.10 | 0.25 ± 0.06 | 0.003 |
| SR cornea | 0.30 ± 0.18 | 0.29 ± 0.13 | 0.713 | 0.35 ± 0.16 | 0.36 ± 0.13 | 0.638 | 0.41 ± 0.09 | 0.39 ± 0.08 | 0.302 |
| Values are presented as mean ± standard deviation or median (interquartile range).  P values were calculated using the independent-samples t test or Mann–Whitney U test, as appropriate. P values less than 0.05 are considered statistically significant.  BCVA, best-corrected visual acuity; DLI, dysfunctional lens index; OSI, objective scatter index; HO, higher-order aberration; MTF, modulation transfer function; TC, total cholesterol. | | | | | | | | | |
|  |  |  |  |  |  |  |  |  |  |
|  |  |  |  |  |  |  |  |  |  |
|  |  |  |  |  |  |  |  |  |  |

| **Supplementary Table S6. Comparison of postoperative visual quality parameters between the low and high TG groups** | | | | | | | | | |
| --- | --- | --- | --- | --- | --- | --- | --- | --- | --- |
|  | 1 day postoperatively | |  | 1 week postoperatively | | | 1 month postoperatively | | |
| Variable | Low TG group (N=55) | High TG group (N=55) | P value | Low TG group (N=55) | High TG group (N=55) | P value | Low TG group (N=55) | High TG group (N=55) | P value |
| DLI | 5.19 ± 1.99 | 5.24 ± 2.26 | 0.903 | 6.53 ± 2.22 | 6.20 ± 2.37 | 0.463 | 7.73 ± 1.70 | 7.34 ± 1.88 | 0.261 |
| OSI | 2.91 ± 1.72 | 2.79 ± 1.52 | 0.686 | 1.69 ± 0.75 | 1.79 ± 0.87 | 0.506 | 1.36 ± 0.38 | 1.38 ± 0.46 | 0.754 |
| BCVA | 0.00 (0.00, 0.10) | 0.00 (0.00, 0.10) | 0.084 | 0.00 (0.00, 0.05) | 0.00 (0.00, 0.00) | 0.714 | 0.00 (0.00, 0.00) | 0.00 (0.00, 0.00) | 0.959 |
| HO total | 0.32 (0.18, 0.46) | 0.32 (0.20, 0.47) | 0.622 | 0.20 (0.14, 0.28) | 0.21 (0.13, 0.32) | 0.652 | 0.15 (0.11, 0.21) | 0.21 (0.14, 0.35) | 0.005 |
| Coma | 0.11 (0.06, 0.18) | 0.13 (0.07, 0.20) | 0.294 | 0.08 (0.05, 0.13) | 0.11 (0.06, 0.17) | 0.131 | 0.07 (0.06, 0.10) | 0.08 (0.06, 0.10) | 0.247 |
| Spherical | 0.03 (0.01, 0.07) | 0.04 (0.02, 0.07) | 0.613 | 0.02 (0.01, 0.04) | 0.02 (0.01, 0.03) | 0.943 | 0.02 (0.01, 0.04) | 0.02 (0.00, 0.03) | 0.071 |
| Secondary Astigmatism | 0.04 (0.02, 0.07) | 0.04 (0.03, 0.08) | 0.482 | 0.04 (0.02, 0.08) | 0.04 (0.02, 0.10) | 0.806 | 0.02 (0.02, 0.03) | 0.03 (0.02, 0.04) | 0.272 |
| Trefoil | 0.18 (0.11, 0.34) | 0.20 (0.13, 0.40) | 0.305 | 0.13 (0.08, 0.20) | 0.15 (0.08, 0.27) | 0.776 | 0.09 (0.07, 0.12) | 0.12 (0.08, 0.17) | 0.043 |
| MTF total eye |  |  |  |  |  |  |  |  |  |
| average height | 0.30 ± 0.09 | 0.32 ± 0.12 | 0.263 | 0.36 ± 0.11 | 0.36 ± 0.11 | 0.928 | 0.41 ± 0.09 | 0.38 ± 0.07 | 0.034 |
| 5 cycles/degree | 0.56 ± 0.17 | 0.58 ± 0.21 | 0.626 | 0.63 ± 0.17 | 0.62 ± 0.19 | 0.765 | 0.71 ± 0.12 | 0.68 ± 0.12 | 0.183 |
| 10 cycles/degree | 0.29 ± 0.14 | 0.33 ± 0.17 | 0.191 | 0.36 ± 0.16 | 0.38 ± 0.16 | 0.562 | 0.45 ± 0.13 | 0.42 ± 0.12 | 0.272 |
| 15 cycles/degree | 0.18 ± 0.10 | 0.21 ± 0.12 | 0.098 | 0.24 ± 0.13 | 0.26 ± 0.14 | 0.363 | 0.30 ± 0.11 | 0.27 ± 0.12 | 0.295 |
| 20 cycles/degree | 0.13 ± 0.07 | 0.15 ± 0.07 | 0.216 | 0.18 ± 0.10 | 0.19 ± 0.11 | 0.414 | 0.23 ± 0.09 | 0.21 ± 0.09 | 0.420 |
| 25 cycles/degree | 0.10 ± 0.05 | 0.11 ± 0.06 | 0.401 | 0.14 ± 0.08 | 0.15 ± 0.09 | 0.384 | 0.17 ± 0.07 | 0.17 ± 0.08 | 0.654 |
| 30 cycles/degree | 0.08 ± 0.04 | 0.09 ± 0.05 | 0.280 | 0.12 ± 0.07 | 0.13 ± 0.07 | 0.438 | 0.15 ± 0.06 | 0.14 ± 0.06 | 0.546 |
| MTF internal |  |  |  |  |  |  |  |  |  |
| average height | 0.34 ± 0.13 | 0.34 ± 0.14 | 0.872 | 0.39 ± 0.13 | 0.37 ± 0.13 | 0.479 | 0.44 ± 0.11 | 0.39 ± 0.11 | 0.025 |
| 5 cycles/degree | 0.60 ± 0.22 | 0.59 ± 0.23 | 0.775 | 0.67 ± 0.19 | 0.65 ± 0.21 | 0.540 | 0.71 ± 0.17 | 0.66 ± 0.18 | 0.124 |
| 10 cycles/degree | 0.35 ± 0.19 | 0.34 ± 0.19 | 0.92 | 0.43 ± 0.18 | 0.41 ± 0.18 | 0.635 | 0.47 ± 0.17 | 0.42 ± 0.16 | 0.128 |
| 15 cycles/degree | 0.22 ± 0.15 | 0.23 ± 0.15 | 0.781 | 0.29 ± 0.15 | 0.27 ± 0.14 | 0.522 | 0.33 ± 0.15 | 0.28 ± 0.13 | 0.055 |
| 20 cycles/degree | 0.16 ± 0.12 | 0.17 ± 0.12 | 0.662 | 0.21 ± 0.12 | 0.21 ± 0.17 | 0.998 | 0.25 ± 0.12 | 0.21 ± 0.10 | 0.049 |
| 25 cycles/degree | 0.14 ± 0.11 | 0.13 ± 0.10 | 0.711 | 0.17 ± 0.10 | 0.16 ± 0.08 | 0.764 | 0.20 ± 0.10 | 0.16 ± 0.08 | 0.046 |
| 30 cycles/degree | 0.11 ± 0.08 | 0.11 ± 0.08 | 0.997 | 0.14 ± 0.09 | 0.13 ± 0.08 | 0.335 | 0.16 ± 0.09 | 0.14 ± 0.07 | 0.067 |
| MTF cornea |  |  |  |  |  |  |  |  |  |
| average height | 0.46 ± 0.14 | 0.47 ± 0.13 | 0.769 | 0.49 ± 0.11 | 0.52 ± 0.11 | 0.284 | 0.55 ± 0.08 | 0.53 ± 0.10 | 0.312 |
| 5 cycles/degree | 0.73 ± 0.15 | 0.73 ± 0.15 | 0.863 | 0.79 ± 0.13 | 0.79 ± 0.13 | 0.867 | 0.82 ± 0.08 | 0.81 ± 0.10 | 0.375 |
| 10 cycles/degree | 0.49 ± 0.18 | 0.52 ± 0.17 | 0.411 | 0.56 ± 0.15 | 0.59 ± 0.14 | 0.295 | 0.62 ± 0.13 | 0.61 ± 0.14 | 0.729 |
| 15 cycles/degree | 0.37 ± 0.18 | 0.38 ± 0.15 | 0.922 | 0.42 ± 0.14 | 0.44 ± 0.13 | 0.597 | 0.46 ± 0.14 | 0.46 ± 0.13 | 0.953 |
| 20 cycles/degree | 0.27 ± 0.13 | 0.28 ± 0.11 | 0.795 | 0.33 ± 0.13 | 0.34 ± 0.12 | 0.592 | 0.38 ± 0.11 | 0.38 ± 0.11 | 0.677 |
| 25 cycles/degree | 0.21 ± 0.10 | 0.20 ± 0.07 | 0.553 | 0.26 ± 0.11 | 0.26 ± 0.10 | 0.901 | 0.30 ± 0.10 | 0.30 ± 0.10 | 0.856 |
| 30 cycles/degree | 0.18 ± 0.08 | 0.17 ± 0.06 | 0.517 | 0.22 ± 0.10 | 0.22 ± 0.09 | 0.791 | 0.26 ± 0.09 | 0.25 ± 0.08 | 0.798 |
| SR total eye | 0.13 ± 0.08 | 0.14 ± 0.09 | 0.354 | 0.19 ± 0.12 | 0.20 ± 0.10 | 0.590 | 0.26 ± 0.09 | 0.22 ± 0.07 | 0.032 |
| SR internal | 0.18 ± 0.15 | 0.18 ± 0.15 | 0.975 | 0.24 ± 0.15 | 0.23 ± 0.12 | 0.705 | 0.30 ± 0.09 | 0.25 ± 0.08 | 0.004 |
| SR cornea | 0.29 ± 0.17 | 0.30 ± 0.15 | 0.820 | 0.35 ± 0.15 | 0.36 ± 0.15 | 0.527 | 0.41 ± 0.09 | 0.40 ± 0.08 | 0.663 |
| Values are presented as mean ± standard deviation or median (interquartile range).  P values were calculated using the independent-samples t test or Mann–Whitney U test, as appropriate. P values less than 0.05 are considered statistically significant.  BCVA, best-corrected visual acuity; DLI, dysfunctional lens index; OSI, objective scatter index; HO, higher-order aberration; MTF, modulation transfer function; TG, triglyceride. | | | | | | | | | |
|  |  |  |  |  |  |  |  |  |  |
|  |  |  |  |  |  |  |  |  |  |
|  |  |  |  |  |  |  |  |  |  |

| **Supplementary Table S7. Comparison of postoperative visual quality parameters between the low and Non-low HDL-C groups** | | | | | | | | | |
| --- | --- | --- | --- | --- | --- | --- | --- | --- | --- |
|  | 1 day postoperatively | |  | 1 week postoperatively | | | 1 month postoperatively | | |
| Variable | Low HDL-C group (N=88) | Non-low HDL-C group (N=22) | P value | Low HDL-C group (N=88) | Non-low HDL-C group (N=22) | P value | Low HDL-C group (N=88) | Non-low HDL-C group (N=22) | P value |
| DLI | 5.26 ± 2.16 | 5.02 ± 1.98 | 0.628 | 6.55 ± 2.30 | 5.61 ± 2.15 | 0.085 | 7.57 ± 1.75 | 7.40 ± 1.97 | 0.689 |
| OSI | 2.94 ± 1.75 | 2.52 ± 0.85 | 0.281 | 1.75 ± 0.85 | 1.72 ± 0.63 | 0.884 | 1.37 ± 0.42 | 1.37 ± 0.46 | 0.991 |
| BCVA | 0.00 (0.00, 0.10) | 0.00 (0.00, 0.10) | 0.946 | 0.00 (0.00, 0.00) | 0.00 (0.00, 0.00) | 0.778 | 0.00 (0.00, 0.00) | 0.00 (0.00, 0.00) | 0.912 |
| HO total | 0.28 (0.18, 0.47) | 0.36 (0.25, 0.44) | 0.390 | 0.20 (0.13, 0.28) | 0.24 (0.18, 0.38) | 0.120 | 0.16 (0.12, 0.24) | 0.19 (0.14, 0.21) | 0.765 |
| Coma | 0.11 (0.06, 0.18) | 0.15 (0.08, 0.24) | 0.173 | 0.08 (0.04, 0.16) | 0.11 (0.07, 0.18) | 0.140 | 0.07 (0.06, 0.09) | 0.10 (0.07, 0.14) | 0.036 |
| Spherical | 0.04 (0.01, 0.07) | 0.04 ± 0.07 | 0.881 | 0.02 (0.01, 0.03) | 0.01 (0.00, 0.02) | 0.227 | 0.02 (0.01, 0.03) | 0.02 (0.01, 0.04) | 0.256 |
| Secondary Astigmatism | 0.04 (0.02, 0.07) | 0.06 (0.03, 0.10) | 0.113 | 0.03 (0.02, 0.08) | 0.05 (0.04, 0.12) | 0.021 | 0.02 (0.02, 0.04) | 0.03 (0.02, 0.03) | 0.748 |
| Trefoil | 0.20 (0.12, 0.37) | 0.21 (0.12, 0.34) | 0.985 | 0.13 (0.08, 0.22) | 0.16 (0.11, 0.23) | 0.322 | 0.10 (0.07, 0.14) | 0.10 (0.08, 0.13) | 0.994 |
| MTF total eye |  |  |  |  |  |  |  |  |  |
| average height | 0.31 ± 0.11 | 0.31 ± 0.10 | 0.907 | 0.36 ± 0.11 | 0.33 ± 0.10 | 0.246 | 0.40 ± 0.08 | 0.40 ± 0.07 | 0.769 |
| 5 cycles/degree | 0.57 ± 0.19 | 0.56 ± 0.19 | 0.785 | 0.63 ± 0.18 | 0.60 ± 0.20 | 0.475 | 0.70 ± 0.12 | 0.69 ± 0.12 | 0.752 |
| 10 cycles/degree | 0.31 ± 0.16 | 0.30 ± 0.16 | 0.856 | 0.38 ± 0.16 | 0.35 ± 0.15 | 0.417 | 0.44 ± 0.13 | 0.41 ± 0.11 | 0.267 |
| 15 cycles/degree | 0.20 ± 0.11 | 0.19 ± 0.11 | 0.868 | 0.26 ± 0.13 | 0.23 ± 0.13 | 0.346 | 0.29 ± 0.12 | 0.26 ± 0.11 | 0.249 |
| 20 cycles/degree | 0.14 ± 0.07 | 0.14 ± 0.08 | 0.670 | 0.19 ± 0.10 | 0.17 ± 0.10 | 0.406 | 0.23 ± 0.09 | 0.19 ± 0.09 | 0.151 |
| 25 cycles/degree | 0.10 ± 0.05 | 0.11 ± 0.06 | 0.587 | 0.15 ± 0.08 | 0.14 ± 0.08 | 0.504 | 0.18 ± 0.07 | 0.16 ± 0.07 | 0.291 |
| 30 cycles/degree | 0.09 ± 0.05 | 0.09 ± 0.05 | 0.632 | 0.12 ± 0.07 | 0.11 ± 0.07 | 0.537 | 0.15 ± 0.06 | 0.13 ± 0.06 | 0.350 |
| MTF internal |  |  |  |  |  |  |  |  |  |
| average height | 0.34 ± 0.13 | 0.33 ± 0.14 | 0.633 | 0.39 ± 0.13 | 0.35 ± 0.10 | 0.217 | 0.41 ± 0.11 | 0.42 ± 0.11 | 0.725 |
| 5 cycles/degree | 0.60 ± 0.22 | 0.56 ± 0.22 | 0.427 | 0.67 ± 0.19 | 0.64 ± 0.21 | 0.645 | 0.70 ± 0.16 | 0.66 ± 0.22 | 0.330 |
| 10 cycles/degree | 0.35 ± 0.19 | 0.32 ± 0.19 | 0.548 | 0.42 ± 0.19 | 0.39 ± 0.16 | 0.440 | 0.45 ± 0.17 | 0.42 ± 0.18 | 0.436 |
| 15 cycles/degree | 0.23 ± 0.15 | 0.21 ± 0.16 | 0.617 | 0.29 ± 0.15 | 0.23 ± 0.11 | 0.137 | 0.31 ± 0.14 | 0.28 ± 0.14 | 0.345 |
| 20 cycles/degree | 0.17 ± 0.12 | 0.16 ± 0.13 | 0.638 | 0.22 ± 0.16 | 0.16 ± 0.08 | 0.082 | 0.23 ± 0.11 | 0.21 ± 0.11 | 0.435 |
| 25 cycles/degree | 0.14 ± 0.10 | 0.13 ± 0.11 | 0.753 | 0.17 ± 0.10 | 0.13 ± 0.05 | 0.027 | 0.18 ± 0.09 | 0.17 ± 0.09 | 0.585 |
| 30 cycles/degree | 0.11 ± 0.08 | 0.11 ± 0.10 | 0.977 | 0.14 ± 0.09 | 0.10 ± 0.05 | 0.045 | 0.15 ± 0.08 | 0.14 ± 0.08 | 0.420 |
| MTF cornea |  |  |  |  |  |  |  |  |  |
| average height | 0.47 ± 0.13 | 0.44 ± 0.13 | 0.423 | 0.50 ± 0.11 | 0.52 ± 0.11 | 0.505 | 0.54 ± 0.10 | 0.55 ± 0.09 | 0.473 |
| 5 cycles/degree | 0.73 ± 0.15 | 0.72 ± 0.13 | 0.706 | 0.78 ± 0.12 | 0.80 ± 0.16 | 0.605 | 0.81 ± 0.10 | 0.83 ± 0.07 | 0.317 |
| 10 cycles/degree | 0.52 ± 0.18 | 0.47 ± 0.16 | 0.232 | 0.57 ± 0.14 | 0.60 ± 0.15 | 0.380 | 0.61 ± 0.14 | 0.62 ± 0.13 | 0.867 |
| 15 cycles/degree | 0.39 ± 0.16 | 0.33 ± 0.14 | 0.144 | 0.43 ± 0.14 | 0.45 ± 0.12 | 0.552 | 0.46 ± 0.13 | 0.47 ± 0.14 | 0.897 |
| 20 cycles/degree | 0.28 ± 0.12 | 0.25 ± 0.11 | 0.206 | 0.33 ± 0.12 | 0.36 ± 0.11 | 0.313 | 0.38 ± 0.11 | 0.40 ± 0.10 | 0.418 |
| 25 cycles/degree | 0.21 ± 0.08 | 0.18 ± 0.08 | 0.152 | 0.26 ± 0.11 | 0.28 ± 0.09 | 0.413 | 0.30 ± 0.10 | 0.32 ± 0.10 | 0.313 |
| 30 cycles/degree | 0.18 ± 0.07 | 0.15 ± 0.08 | 0.168 | 0.22 ± 0.10 | 0.23 ± 0.08 | 0.547 | 0.25 ± 0.08 | 0.27 ± 0.09 | 0.321 |
| SR total eye | 0.14 ± 0.09 | 0.14 ± 0.09 | 0.990 | 0.20 ± 0.11 | 0.17 ± 0.09 | 0.336 | 0.24 ± 0.09 | 0.22 ± 0.06 | 0.228 |
| SR internal | 0.18 ± 0.14 | 0.17 ± 0.17 | 0.756 | 0.25 ± 0.15 | 0.19 ± 0.09 | 0.069 | 0.28 ± 0.09 | 0.26 ± 0.08 | 0.397 |
| SR cornea | 0.31 ± 0.16 | 0.26 ± 0.15 | 0.231 | 0.35 ± 0.15 | 0.37 ± 0.13 | 0.564 | 0.41 ± 0.08 | 0.39 ± 0.08 | 0.466 |
| Values are presented as mean ± standard deviation or median (interquartile range).  P values were calculated using the independent-samples t test or Mann–Whitney U test, as appropriate. P values less than 0.05 are considered statistically significant.  BCVA, best-corrected visual acuity; DLI, dysfunctional lens index; OSI, objective scatter index; HO, higher-order aberration; MTF, modulation transfer function; HDL-C, high-density lipoprotein cholesterol. | | | | | | | | | |
|  |  |  |  |  |  |  |  |  |  |
|  |  |  |  |  |  |  |  |  |  |
|  |  |  |  |  |  |  |  |  |  |

| **Supplementary Table S8. Comparison of postoperative visual quality parameters between the at-target and above-target LDL-C groups** | | | | | | | | | |
| --- | --- | --- | --- | --- | --- | --- | --- | --- | --- |
|  | 1 day postoperatively | |  | 1 week postoperatively | | | 1 month postoperatively | | |
| Variable | at-target LDL-C group (N=60) | above-target LDL-C group (N=50) | P value | at-target LDL-C group (N=60) | above-target LDL-C group (N=50) | P value | at-target LDL-C group (N=60) | above-target LDL-C group (N=50) | P value |
| DLI | 5.36 ± 1.90 | 5.03 ± 2.36 | 0.415 | 6.85 ± 2.11 | 5.78 ± 2.38 | 0.014 | 7.79 ± 1.65 | 7.23 ± 1.92 | 0.099 |
| OSI | 2.73 ± 1.60 | 3.00 ± 1.64 | 0.389 | 1.68 ± 0.82 | 1.81 ± 0.80 | 0.391 | 1.29 ± 0.37 | 1.46 ± 0.46 | 0.039 |
| BCVA | 0.00 (0.00, 0.10) | 0.00 (0.00, 0.10) | 0.298 | 0.00 (0.00, 0.00) | 0.00 (0.00, 0.00) | 0.789 | 0.00 (0.00, 0.00) | 0.00 (0.00, 0.00) | 0.637 |
| HO total | 0.32 (0.20, 0.45) | 0.30 (0.18, 0.52) | 0.817 | 0.19 (0.14, 0.27) | 0.23 (0.16, 0.34) | 0.134 | 0.16 ± 0.05 | 0.21 (0.12, 0.37) | 0.005 |
| Coma | 0.11 (0.06, 0.18) | 0.13 (0.07, 0.19) | 0.448 | 0.08 (0.05, 0.13) | 0.10 (0.05, 0.17) | 0.168 | 0.08 (0.06, 0.11) | 0.08 (0.06, 0.10) | 0.468 |
| Spherical | 0.03 (0.01, 0.07) | 0.04 (0.02, 0.08) | 0.215 | 0.02 (0.01, 0.04) | 0.02 (0.01, 0.03) | 0.874 | 0.02 (0.01, 0.04) | 0.02 (0.01, 0.03) | 0.216 |
| Secondary Astigmatism | 0.04 (0.02, 0.07) | 0.05 (0.03, 0.09) | 0.253 | 0.03 (0.02, 0.07) | 0.04 (0.02, 0.12) | 0.129 | 0.02 (0.02, 0.03) | 0.03 (0.02, 0.04) | 0.059 |
| Trefoil | 0.19 (0.12, 0.33) | 0.20 (0.12, 0.40) | 0.661 | 0.12 (0.08, 0.20) | 0.15 (0.09, 0.27) | 0.163 | 0.09 (0.07, 0.14) | 0.11 (0.08, 0.14) | 0.202 |
| MTF total eye |  |  |  |  |  |  |  |  |  |
| average height | 0.32 ± 0.09 | 0.31 ± 0.12 | 0.592 | 0.37 ± 0.11 | 0.33 ± 0.11 | 0.054 | 0.42 ± 0.09 | 0.38 ± 0.07 | 0.013 |
| 5 cycles/degree | 0.58 ± 0.16 | 0.55 ± 0.22 | 0.384 | 0.66 ± 0.17 | 0.59 ± 0.19 | 0.063 | 0.72 ± 0.12 | 0.68 ± 0.11 | 0.083 |
| 10 cycles/degree | 0.31 ± 0.14 | 0.30 ± 0.17 | 0.758 | 0.40 ± 0.16 | 0.35 ± 0.16 | 0.095 | 0.45 ± 0.13 | 0.41 ± 0.11 | 0.066 |
| 15 cycles/degree | 0.19 ± 0.10 | 0.19 ± 0.13 | 0.993 | 0.26 ± 0.13 | 0.23 ± 0.13 | 0.239 | 0.30 ± 0.11 | 0.26 ± 0.11 | 0.101 |
| 20 cycles/degree | 0.14 ± 0.07 | 0.13 ± 0.07 | 0.378 | 0.19 ± 0.10 | 0.17 ± 0.10 | 0.278 | 0.23 ± 0.09 | 0.21 ± 0.09 | 0.250 |
| 25 cycles/degree | 0.11 ± 0.05 | 0.10 ± 0.05 | 0.169 | 0.15 ± 0.08 | 0.14 ± 0.08 | 0.347 | 0.18 ± 0.07 | 0.16 ± 0.07 | 0.347 |
| 30 cycles/degree | 0.09 ± 0.04 | 0.08 ± 0.05 | 0.383 | 0.13 ± 0.07 | 0.11 ± 0.07 | 0.319 | 0.15 ± 0.06 | 0.14 ± 0.06 | 0.296 |
| MTF internal |  |  |  |  |  |  |  |  |  |
| average height | 0.35 ± 0.13 | 0.32 ± 0.14 | 0.159 | 0.41 ± 0.12 | 0.35 ± 0.12 | 0.011 | 0.44 ± 0.11 | 0.38 ± 0.11 | 0.001 |
| 5 cycles/degree | 0.62 ± 0.20 | 0.56 ± 0.24 | 0.167 | 0.70 ± 0.17 | 0.61 ± 0.21 | 0.022 | 0.72 ± 0.17 | 0.65 ± 0.18 | 0.034 |
| 10 cycles/degree | 0.37 ± 0.19 | 0.32 ± 0.20 | 0.182 | 0.45 ± 0.17 | 0.37 ± 0.18 | 0.016 | 0.48 ± 0.17 | 0.41 ± 0.16 | 0.027 |
| 15 cycles/degree | 0.24 ± 0.15 | 0.21 ± 0.15 | 0.263 | 0.31 ± 0.15 | 0.24 ± 0.13 | 0.013 | 0.33 ± 0.15 | 0.27 ± 0.13 | 0.015 |
| 20 cycles/degree | 0.18 ± 0.12 | 0.16 ± 0.12 | 0.431 | 0.23 ± 0.13 | 0.19 ± 0.17 | 0.191 | 0.25 ± 0.12 | 0.20 ± 0.10 | 0.014 |
| 25 cycles/degree | 0.14 ± 0.10 | 0.13 ± 0.11 | 0.571 | 0.18 ± 0.10 | 0.15 ± 0.07 | 0.057 | 0.20 ± 0.10 | 0.16 ± 0.08 | 0.017 |
| 30 cycles/degree | 0.12 ± 0.08 | 0.10 ± 0.08 | 0.228 | 0.15 ± 0.09 | 0.11 ± 0.07 | 0.013 | 0.17 ± 0.09 | 0.13 ± 0.07 | 0.026 |
| MTF cornea |  |  |  |  |  |  |  |  |  |
| average height | 0.46 ± 0.14 | 0.46 ± 0.13 | 0.896 | 0.50 ± 0.12 | 0.51 ± 0.09 | 0.728 | 0.56 ± 0.07 | 0.51 ± 0.11 | 0.003 |
| 5 cycles/degree | 0.74 ± 0.15 | 0.72 ± 0.15 | 0.629 | 0.79 ± 0.13 | 0.79 ± 0.13 | 0.888 | 0.83 ± 0.07 | 0.79 ± 0.11 | 0.008 |
| 10 cycles/degree | 0.51 ± 0.19 | 0.51 ± 0.17 | 0.988 | 0.57 ± 0.16 | 0.59 ± 0.13 | 0.520 | 0.64 ± 0.12 | 0.58 ± 0.15 | 0.036 |
| 15 cycles/degree | 0.38 ± 0.17 | 0.37 ± 0.15 | 0.718 | 0.43 ± 0.15 | 0.43 ± 0.11 | 0.981 | 0.48 ± 0.13 | 0.44 ± 0.13 | 0.134 |
| 20 cycles/degree | 0.29 ± 0.13 | 0.26 ± 0.10 | 0.311 | 0.34 ± 0.14 | 0.34 ± 0.09 | 0.958 | 0.39 ± 0.10 | 0.36 ± 0.12 | 0.135 |
| 25 cycles/degree | 0.21 ± 0.09 | 0.19 ± 0.07 | 0.222 | 0.27 ± 0.12 | 0.26 ± 0.08 | 0.540 | 0.32 ± 0.09 | 0.28 ± 0.10 | 0.056 |
| 30 cycles/degree | 0.18 ± 0.08 | 0.16 ± 0.06 | 0.108 | 0.23 ± 0.11 | 0.21 ± 0.07 | 0.356 | 0.27 ± 0.08 | 0.24 ± 0.09 | 0.103 |
| SR total eye | 0.14 ± 0.08 | 0.13 ± 0.09 | 0.463 | 0.21 ± 0.12 | 0.17 ± 0.09 | 0.135 | 0.26 ± 0.09 | 0.22 ± 0.07 | 0.009 |
| SR internal | 0.19 ± 0.15 | 0.16 ± 0.15 | 0.308 | 0.26 ± 0.15 | 0.21 ± 0.11 | 0.046 | 0.30 ± 0.10 | 0.25 ± 0.06 | 0.002 |
| SR cornea | 0.30 ± 0.17 | 0.29 ± 0.14 | 0.751 | 0.36 ± 0.18 | 0.35 ± 0.10 | 0.803 | 0.41 ± 0.09 | 0.39 ± 0.08 | 0.195 |
| Values are presented as mean ± standard deviation or median (interquartile range).  P values were calculated using the independent-samples t test or Mann–Whitney U test, as appropriate. P values less than 0.05 are considered statistically significant.  BCVA, best-corrected visual acuity; DLI, dysfunctional lens index; OSI, objective scatter index; HO, higher-order aberration; MTF, modulation transfer function; LDL-C, low-density lipoprotein cholesterol. | | | | | | | | | |
|  |  |  |  |  |  |  |  |  |  |
|  |  |  |  |  |  |  |  |  |  |
|  |  |  |  |  |  |  |  |  |  |

| **Supplementary Table S9. Spearman correlations between preoperative lipid parameters and postoperative month 1 visual quality indices** | | | | | | | | | | | | | | |
| --- | --- | --- | --- | --- | --- | --- | --- | --- | --- | --- | --- | --- | --- | --- |
|  |  | HO total | DLI | OSI | MTF total eye average height | MTF internal average height | MTF cornea average height | SR total eye | SR internal | SR cornea | TG | TC | HDL-C | LDL-C |
| HO total | r | - |  |  |  |  |  |  |  |  |  |  |  |  |
|  | P value | - |  |  |  |  |  |  |  |  |  |  |  |  |
| DLI | r | -.236* | - |  |  |  |  |  |  |  |  |  |  |  |
|  | P value | 0.013 | - |  |  |  |  |  |  |  |  |  |  |  |
| OSI | r | 0.05 | -0.16 | - |  |  |  |  |  |  |  |  |  |  |
|  | P value | 0.585 | 0.1 | - |  |  |  |  |  |  |  |  |  |  |
| MTF total eye average height | r | -0.30 | 0.39 | 0.01 | - |  |  |  |  |  |  |  |  |  |
|  | P value | 0.001 | <.001 | 0.928 | - |  |  |  |  |  |  |  |  |  |
| MTF internal average height | r | -0.29 | 0.46 | -0.12 | 0.57 | - |  |  |  |  |  |  |  |  |
|  | P value | 0.002 | <.001 | 0.197 | <.001 | - |  |  |  |  |  |  |  |  |
| MTF cornea average height | r | -0.02 | -0.01 | -0.26 | 0.23 | 0.41 | - |  |  |  |  |  |  |  |
|  | P value | 0.837 | 0.914 | 0.006 | 0.017 | <.001 | - |  |  |  |  |  |  |  |
| SR total eye | r | -0.42 | 0.35 | -0.16 | 0.62 | 0.49 | 0.23 | - |  |  |  |  |  |  |
|  | P value | <.001 | <.001 | 0.1 | <.001 | <.001 | 0.016 | - |  |  |  |  |  |  |
| SR internal | r | -0.22 | 0.24 | -0.15 | 0.43 | 0.56 | 0.31 | 0.33 | - |  |  |  |  |  |
|  | P value | 0.021 | 0.012 | 0.131 | <.001 | <.001 | 0.001 | <.001 | - |  |  |  |  |  |
| SR cornea | r | -0.08 | 0.03 | -0.24 | 0.25 | 0.28 | 0.63 | 0.36 | 0.31 | - |  |  |  |  |
|  | P value | 0.39 | 0.724 | 0.01 | 0.009 | 0.004 | <.001 | <.001 | <.001 | - |  |  |  |  |
| TG | r | 0.11 | -0.13 | 0.04 | -0.29 | -0.31 | -0.10 | -0.25 | -0.33 | -0.12 | - |  |  |  |
|  | P value | 0.238 | 0.175 | 0.656 | 0.002 | 0.001 | 0.301 | 0.008 | <.001 | 0.218 | - |  |  |  |
| TC | r | 0.17 | -0.06 | -0.05 | -0.21 | -0.18 | -0.13 | -0.26 | -0.25 | -0.07 | 0.74 | - |  |  |
|  | P value | 0.078 | 0.554 | 0.631 | 0.032 | 0.067 | 0.194 | 0.007 | 0.01 | 0.483 | <.001 | - |  |  |
| HDL-C | r | -0.01 | 0.03 | -0.06 | 0.05 | 0.06 | 0.14 | -0.02 | 0.03 | 0.09 | -0.21 | 0.13 | - |  |
|  | P value | 0.942 | 0.758 | 0.519 | 0.624 | 0.507 | 0.136 | 0.825 | 0.755 | 0.364 | 0.031 | 0.187 | - |  |
| LDL-C | r | 0.24 | -0.12 | -0.03 | -0.24 | -0.22 | -0.17 | -0.22 | -0.24 | -0.03 | 0.53 | 0.82 | 0.08 | - |
|  | P value | 0.01 | 0.222 | 0.787 | 0.012 | 0.023 | 0.082 | 0.023 | 0.011 | 0.76 | <.001 | <.001 | 0.409 | - |
| Data are presented as Spearman correlation coefficients (r) and corresponding two-sided P values. All correlations were calculated using postoperative month-1 visual quality parameters and preoperative lipid levels. N = 110 for all analyses. TC, total cholesterol; TG, triglycerides; HDL-C, high-density lipoprotein cholesterol; LDL-C, low-density lipoprotein cholesterol; DLI, dysfunctional lens index; OSI, objective scatter index; HO, higher-order aberration; MTF, modulation transfer function; SR, Strehl ratio. | | | | | | | | | | | | | | |
|  |  |  |  |  |  |  |  |  |  |  |  |  |  |  |
|  |  |  |  |  |  |  |  |  |  |  |  |  |  |  |

| **Supplementary Table S10. Longitudinal associations of four lipid parameters with postoperative visual outcomes in linear mixed-effects models** | | | | | | | | | | | |
| --- | --- | --- | --- | --- | --- | --- | --- | --- | --- | --- | --- |
| Outcome | Lipid parameter | POD1 slope, β (95% CI) | P value | POW1 slope, β (95% CI) | P value | POM1 slope, β (95% CI) | P value | POW1 vs POD1 interaction to β (95% CI) | P value | POM1 vs POD1 interaction to β (95% CI) | P value |
| BCVA | LDL-C | -0.046 (-0.069 to -0.023) | 0.002 | -0.005 (-0.028 to 0.018) | 0.765 | -0.013 (-0.036 to 0.010) | 0.919 | 0.042 (0.021 to 0.062) | 0.003 | 0.033 (0.012 to 0.054) | 0.017 |
| Coma | LDL-C | 0.001 (-0.032 to 0.034) | 0.967 | 0.006 (-0.027 to 0.039) | 0.765 | -0.016 (-0.049 to 0.017) | 0.919 | 0.005 (-0.026 to 0.037) | 0.860 | -0.017 (-0.048 to 0.014) | 0.698 |
| DLI | LDL-C | 0.079 (-0.499 to 0.657) | 0.967 | -0.231 (-0.809 to 0.347) | 0.765 | 0.030 (-0.549 to 0.608) | 0.919 | -0.310 (-0.762 to 0.142) | 0.554 | -0.049 (-0.502 to 0.403) | 0.860 |
| HO total | LDL-C | 0.042 (-0.066 to 0.150) | 0.929 | 0.029 (-0.079 to 0.137) | 0.765 | 0.016 (-0.092 to 0.125) | 0.919 | -0.014 (-0.104 to 0.076) | 0.860 | -0.026 (-0.116 to 0.064) | 0.728 |
| MTF cornea average height | LDL-C | -0.001 (-0.028 to 0.027) | 0.967 | -0.005 (-0.033 to 0.023) | 0.765 | -0.014 (-0.042 to 0.014) | 0.919 | -0.005 (-0.037 to 0.028) | 0.860 | -0.014 (-0.046 to 0.019) | 0.698 |
| MTF internal average height | LDL-C | -0.001 (-0.035 to 0.033) | 0.967 | -0.020 (-0.054 to 0.014) | 0.765 | -0.011 (-0.045 to 0.023) | 0.919 | -0.019 (-0.045 to 0.008) | 0.554 | -0.010 (-0.036 to 0.017) | 0.698 |
| MTF total eye average height | LDL-C | 0.012 (-0.015 to 0.039) | 0.929 | -0.010 (-0.037 to 0.017) | 0.765 | -0.008 (-0.035 to 0.019) | 0.919 | -0.022 (-0.045 to 0.001) | 0.317 | -0.020 (-0.043 to 0.003) | 0.409 |
| OSI | LDL-C | -0.113 (-0.388 to 0.161) | 0.929 | 0.008 (-0.267 to 0.282) | 0.955 | -0.016 (-0.290 to 0.259) | 0.919 | 0.121 (-0.193 to 0.435) | 0.698 | 0.098 (-0.216 to 0.412) | 0.720 |
| SR cornea | LDL-C | -0.007 (-0.041 to 0.027) | 0.967 | -0.021 (-0.055 to 0.014) | 0.765 | 0.006 (-0.029 to 0.040) | 0.919 | -0.014 (-0.052 to 0.024) | 0.698 | 0.013 (-0.026 to 0.051) | 0.720 |
| SR internal | LDL-C | 0.001 (-0.032 to 0.034) | 0.967 | -0.015 (-0.047 to 0.018) | 0.765 | -0.014 (-0.047 to 0.019) | 0.919 | -0.016 (-0.049 to 0.017) | 0.698 | -0.015 (-0.048 to 0.018) | 0.698 |
| SR total eye | LDL-C | 0.008 (-0.017 to 0.032) | 0.929 | -0.005 (-0.029 to 0.020) | 0.765 | -0.001 (-0.026 to 0.023) | 0.919 | -0.013 (-0.036 to 0.011) | 0.698 | -0.009 (-0.033 to 0.014) | 0.698 |
| Secondary astigmatism | LDL-C | 0.010 (-0.018 to 0.038) | 0.929 | 0.010 (-0.018 to 0.038) | 0.765 | -0.009 (-0.037 to 0.019) | 0.919 | -0.000 (-0.026 to 0.026) | 0.999 | -0.019 (-0.045 to 0.007) | 0.554 |
| Spherical | LDL-C | 0.017 (0.003 to 0.032) | 0.135 | -0.007 (-0.022 to 0.007) | 0.765 | -0.013 (-0.028 to 0.001) | 0.919 | -0.024 (-0.042 to -0.007) | 0.050 | -0.030 (-0.048 to -0.013) | 0.012 |
| Trefoil | LDL-C | 0.023 (-0.041 to 0.086) | 0.929 | 0.017 (-0.047 to 0.080) | 0.765 | 0.003 (-0.060 to 0.067) | 0.919 | -0.006 (-0.055 to 0.043) | 0.860 | -0.019 (-0.069 to 0.030) | 0.698 |
| BCVA | HDL-C | -0.034 (-0.095 to 0.027) | 0.736 | -0.018 (-0.080 to 0.043) | 0.722 | -0.027 (-0.088 to 0.034) | 0.963 | 0.016 (-0.048 to 0.079) | 0.804 | 0.007 (-0.056 to 0.070) | 0.963 |
| Coma | HDL-C | 0.019 (-0.066 to 0.103) | 0.830 | 0.134 (0.049 to 0.218) | 0.029 | 0.022 (-0.063 to 0.106) | 0.963 | 0.115 (0.025 to 0.205) | 0.116 | 0.003 (-0.087 to 0.093) | 0.970 |
| DLI | HDL-C | 0.167 (-1.308 to 1.642) | 0.830 | -0.905 (-2.380 to 0.570) | 0.627 | 0.529 (-0.946 to 2.004) | 0.963 | -1.072 (-2.389 to 0.245) | 0.438 | 0.362 (-0.955 to 1.679) | 0.804 |
| HO total | HDL-C | -0.134 (-0.410 to 0.143) | 0.736 | 0.213 (-0.064 to 0.489) | 0.513 | -0.070 (-0.347 to 0.206) | 0.963 | 0.346 (0.087 to 0.605) | 0.116 | 0.063 (-0.196 to 0.322) | 0.804 |
| MTF cornea average height | HDL-C | -0.053 (-0.127 to 0.022) | 0.575 | 0.022 (-0.053 to 0.096) | 0.722 | 0.004 (-0.071 to 0.078) | 0.963 | 0.074 (-0.021 to 0.170) | 0.438 | 0.057 (-0.039 to 0.152) | 0.510 |
| MTF internal average height | HDL-C | 0.009 (-0.077 to 0.096) | 0.830 | -0.026 (-0.113 to 0.060) | 0.722 | 0.049 (-0.038 to 0.135) | 0.963 | -0.036 (-0.114 to 0.042) | 0.548 | 0.039 (-0.039 to 0.117) | 0.538 |
| MTF total eye average height | HDL-C | 0.022 (-0.048 to 0.092) | 0.828 | -0.012 (-0.082 to 0.059) | 0.743 | 0.021 (-0.049 to 0.091) | 0.963 | -0.034 (-0.102 to 0.034) | 0.538 | -0.001 (-0.069 to 0.067) | 0.970 |
| OSI | HDL-C | -0.747 (-1.476 to -0.019) | 0.575 | -0.122 (-0.851 to 0.606) | 0.743 | -0.216 (-0.945 to 0.512) | 0.963 | 0.625 (-0.292 to 1.542) | 0.473 | 0.531 (-0.386 to 1.448) | 0.510 |
| SR cornea | HDL-C | -0.070 (-0.161 to 0.021) | 0.575 | 0.020 (-0.072 to 0.111) | 0.743 | 0.005 (-0.086 to 0.097) | 0.963 | 0.090 (-0.022 to 0.201) | 0.438 | 0.075 (-0.036 to 0.187) | 0.473 |
| SR internal | HDL-C | 0.014 (-0.071 to 0.100) | 0.830 | -0.063 (-0.149 to 0.022) | 0.513 | -0.002 (-0.088 to 0.084) | 0.963 | -0.078 (-0.174 to 0.019) | 0.438 | -0.016 (-0.113 to 0.080) | 0.896 |
| SR total eye | HDL-C | 0.024 (-0.039 to 0.088) | 0.792 | -0.024 (-0.087 to 0.040) | 0.722 | -0.010 (-0.074 to 0.054) | 0.963 | -0.048 (-0.116 to 0.020) | 0.473 | -0.035 (-0.103 to 0.034) | 0.538 |
| Secondary astigmatism | HDL-C | -0.011 (-0.084 to 0.062) | 0.830 | 0.072 (-0.001 to 0.145) | 0.372 | -0.005 (-0.078 to 0.068) | 0.963 | 0.083 (0.007 to 0.159) | 0.224 | 0.006 (-0.070 to 0.082) | 0.970 |
| Spherical | HDL-C | -0.018 (-0.058 to 0.021) | 0.736 | -0.021 (-0.061 to 0.018) | 0.627 | 0.006 (-0.033 to 0.046) | 0.963 | -0.003 (-0.056 to 0.050) | 0.970 | 0.024 (-0.029 to 0.077) | 0.548 |
| Trefoil | HDL-C | -0.120 (-0.281 to 0.042) | 0.575 | 0.083 (-0.079 to 0.244) | 0.627 | -0.034 (-0.196 to 0.127) | 0.963 | 0.203 (0.060 to 0.345) | 0.116 | 0.086 (-0.056 to 0.228) | 0.510 |
| BCVA | TC | -0.022 (-0.041 to -0.003) | 0.351 | -0.003 (-0.022 to 0.017) | 0.913 | -0.009 (-0.029 to 0.010) | 0.882 | 0.019 (0.002 to 0.037) | 0.301 | 0.013 (-0.005 to 0.030) | 0.611 |
| Coma | TC | 0.000 (-0.027 to 0.027) | 0.988 | 0.013 (-0.014 to 0.040) | 0.913 | -0.013 (-0.040 to 0.014) | 0.882 | 0.013 (-0.013 to 0.038) | 0.712 | -0.014 (-0.039 to 0.012) | 0.712 |
| DLI | TC | 0.148 (-0.325 to 0.621) | 0.921 | -0.099 (-0.572 to 0.375) | 0.913 | 0.188 (-0.286 to 0.661) | 0.882 | -0.247 (-0.622 to 0.128) | 0.611 | 0.040 (-0.335 to 0.415) | 0.899 |
| HO total | TC | 0.012 (-0.077 to 0.101) | 0.921 | 0.026 (-0.063 to 0.114) | 0.913 | -0.011 (-0.100 to 0.078) | 0.882 | 0.014 (-0.061 to 0.088) | 0.860 | -0.023 (-0.097 to 0.052) | 0.860 |
| MTF cornea average height | TC | 0.004 (-0.019 to 0.027) | 0.921 | 0.009 (-0.014 to 0.032) | 0.913 | -0.003 (-0.026 to 0.020) | 0.882 | 0.005 (-0.022 to 0.033) | 0.860 | -0.006 (-0.034 to 0.021) | 0.860 |
| MTF internal average height | TC | 0.005 (-0.023 to 0.033) | 0.921 | -0.005 (-0.033 to 0.022) | 0.913 | -0.001 (-0.029 to 0.027) | 0.937 | -0.010 (-0.032 to 0.012) | 0.716 | -0.006 (-0.028 to 0.016) | 0.860 |
| MTF total eye average height | TC | 0.013 (-0.009 to 0.036) | 0.921 | 0.004 (-0.018 to 0.026) | 0.913 | -0.003 (-0.025 to 0.019) | 0.882 | -0.010 (-0.029 to 0.010) | 0.712 | -0.016 (-0.036 to 0.003) | 0.611 |
| OSI | TC | -0.114 (-0.339 to 0.112) | 0.921 | 0.002 (-0.224 to 0.227) | 0.989 | -0.051 (-0.277 to 0.175) | 0.882 | 0.115 (-0.145 to 0.376) | 0.716 | 0.063 (-0.198 to 0.323) | 0.860 |
| SR cornea | TC | 0.005 (-0.024 to 0.033) | 0.921 | 0.006 (-0.022 to 0.034) | 0.913 | 0.009 (-0.020 to 0.037) | 0.882 | 0.001 (-0.030 to 0.033) | 0.930 | 0.004 (-0.028 to 0.036) | 0.899 |
| SR internal | TC | 0.007 (-0.020 to 0.034) | 0.921 | -0.008 (-0.035 to 0.018) | 0.913 | -0.011 (-0.038 to 0.016) | 0.882 | -0.016 (-0.043 to 0.012) | 0.712 | -0.018 (-0.046 to 0.009) | 0.611 |
| SR total eye | TC | 0.008 (-0.013 to 0.028) | 0.921 | 0.002 (-0.019 to 0.022) | 0.945 | -0.006 (-0.026 to 0.014) | 0.882 | -0.006 (-0.025 to 0.013) | 0.860 | -0.014 (-0.033 to 0.006) | 0.611 |
| Secondary astigmatism | TC | 0.007 (-0.016 to 0.030) | 0.921 | 0.009 (-0.014 to 0.032) | 0.913 | -0.008 (-0.031 to 0.015) | 0.882 | 0.002 (-0.020 to 0.023) | 0.906 | -0.015 (-0.037 to 0.006) | 0.611 |
| Spherical | TC | 0.008 (-0.004 to 0.020) | 0.921 | -0.009 (-0.021 to 0.003) | 0.913 | -0.014 (-0.026 to -0.002) | 0.344 | -0.017 (-0.032 to -0.002) | 0.301 | -0.022 (-0.037 to -0.007) | 0.105 |
| Trefoil | TC | -0.000 (-0.052 to 0.052) | 0.988 | 0.008 (-0.044 to 0.060) | 0.913 | -0.007 (-0.059 to 0.045) | 0.882 | 0.008 (-0.033 to 0.049) | 0.860 | -0.007 (-0.048 to 0.034) | 0.860 |
| BCVA | TG | 0.011 (0.003 to 0.019) | 0.112 | 0.003 (-0.005 to 0.011) | 0.564 | 0.004 (-0.004 to 0.012) | 0.650 | -0.008 (-0.016 to 0.000) | 0.420 | -0.007 (-0.015 to 0.001) | 0.420 |
| Coma | TG | -0.005 (-0.016 to 0.007) | 0.951 | -0.009 (-0.020 to 0.002) | 0.161 | -0.008 (-0.019 to 0.003) | 0.650 | -0.004 (-0.016 to 0.007) | 0.675 | -0.004 (-0.016 to 0.008) | 0.675 |
| DLI | TG | 0.060 (-0.133 to 0.253) | 0.951 | 0.120 (-0.073 to 0.313) | 0.282 | 0.036 (-0.157 to 0.229) | 0.767 | 0.060 (-0.110 to 0.231) | 0.675 | -0.023 (-0.194 to 0.147) | 0.919 |
| HO total | TG | -0.018 (-0.054 to 0.018) | 0.951 | -0.030 (-0.066 to 0.006) | 0.154 | -0.015 (-0.051 to 0.021) | 0.650 | -0.013 (-0.046 to 0.021) | 0.675 | 0.002 (-0.031 to 0.036) | 0.962 |
| MTF cornea average height | TG | -0.000 (-0.010 to 0.010) | 0.989 | 0.011 (0.001 to 0.020) | 0.103 | 0.005 (-0.005 to 0.014) | 0.650 | 0.011 (-0.001 to 0.023) | 0.420 | 0.005 (-0.008 to 0.017) | 0.675 |
| MTF internal average height | TG | 0.002 (-0.010 to 0.013) | 0.973 | 0.011 (-0.000 to 0.022) | 0.126 | -0.002 (-0.013 to 0.009) | 0.767 | 0.009 (-0.001 to 0.019) | 0.420 | -0.004 (-0.014 to 0.006) | 0.675 |
| MTF total eye average height | TG | 0.004 (-0.005 to 0.013) | 0.951 | 0.010 (0.001 to 0.019) | 0.103 | -0.003 (-0.012 to 0.006) | 0.650 | 0.007 (-0.002 to 0.015) | 0.460 | -0.007 (-0.015 to 0.002) | 0.453 |
| OSI | TG | -0.032 (-0.128 to 0.063) | 0.951 | 0.005 (-0.090 to 0.101) | 0.914 | -0.000 (-0.096 to 0.095) | 0.992 | 0.038 (-0.080 to 0.156) | 0.675 | 0.032 (-0.086 to 0.150) | 0.724 |
| SR cornea | TG | -0.000 (-0.012 to 0.012) | 0.989 | 0.017 (0.005 to 0.029) | 0.065 | 0.004 (-0.007 to 0.016) | 0.650 | 0.017 (0.003 to 0.031) | 0.420 | 0.005 (-0.010 to 0.019) | 0.675 |
| SR internal | TG | 0.000 (-0.011 to 0.012) | 0.989 | 0.009 (-0.002 to 0.021) | 0.154 | -0.004 (-0.015 to 0.007) | 0.650 | 0.009 (-0.003 to 0.021) | 0.464 | -0.004 (-0.016 to 0.008) | 0.675 |
| SR total eye | TG | 0.002 (-0.007 to 0.010) | 0.973 | 0.009 (0.001 to 0.017) | 0.103 | -0.003 (-0.012 to 0.005) | 0.650 | 0.007 (-0.001 to 0.016) | 0.420 | -0.005 (-0.014 to 0.004) | 0.675 |
| Secondary astigmatism | TG | -0.006 (-0.015 to 0.004) | 0.951 | -0.010 (-0.020 to -0.001) | 0.103 | -0.006 (-0.015 to 0.004) | 0.650 | -0.005 (-0.014 to 0.005) | 0.675 | -0.000 (-0.010 to 0.009) | 0.971 |
| Spherical | TG | -0.001 (-0.006 to 0.004) | 0.973 | -0.001 (-0.006 to 0.004) | 0.748 | -0.004 (-0.009 to 0.001) | 0.650 | -0.000 (-0.007 to 0.007) | 0.971 | -0.003 (-0.010 to 0.004) | 0.675 |
| Trefoil | TG | -0.010 (-0.031 to 0.011) | 0.951 | -0.018 (-0.039 to 0.003) | 0.154 | -0.009 (-0.030 to 0.012) | 0.650 | -0.008 (-0.026 to 0.011) | 0.675 | 0.001 (-0.017 to 0.020) | 0.962 |
| Data are presented as regression coefficients (β) with 95% confidence intervals (CIs). Postoperative day 1 (POD1) was treated as the reference time point. The POD1, POW1, and POM1 slopes represent the associations LDL-C and the outcomes at POD1, POW1, and POM1, respectively. Interaction terms indicate the change in the LDL-C slope at POW1 or POM1 relative to POD1. Models were adjusted for age, body mass index, preoperative BCVA, axial length, corneal astigmatism, sex, diabetes, hypertension, and smoking. P values were adjusted for multiple comparisons using the Benjamini-Hochberg false discovery rate method. | | | | | | | | | | | |
|  |  |  |  |  |  |  |  |  |  |  |  |
|  |  |  |  |  |  |  |  |  |  |  |  |
|  |  |  |  |  |  |  |  |  |  |  |  |
|  |  |  |  |  |  |  |  |  |  |  |  |
|  |  |  |  |  |  |  |  |  |  |  |  |

| **Supplementary Table S11. Sensitivity analysis of longitudinal associations between HDL-C and postoperative visual outcomes after additional adjustment for marital status and IOL power** | | | | | |
| --- | --- | --- | --- | --- | --- |
| Outcome | Effect | Original β (95% CI) | Original FDR P value | Sensitivity β (95% CI) | Sensitivity FDR P value |
| BCVA | POD1 slope | -0.034 (-0.095 to 0.027) | 0.601 | -0.023 (-0.089 to 0.043) | 0.718 |
| BCVA | POW1 vs POD1 | 0.016 (-0.048 to 0.079) | 0.856 | 0.016 (-0.048 to 0.079) | 0.804 |
| BCVA | POM1 vs POD1 | 0.007 (-0.056 to 0.070) | 0.918 | 0.007 (-0.056 to 0.070) | 0.912 |
| Coma | POD1 slope | 0.019 (-0.066 to 0.103) | 0.871 | -0.015 (-0.102 to 0.072) | 0.836 |
| Coma | POW1 vs POD1 | 0.115 (0.025 to 0.205) | 0.174 | 0.115 (0.025 to 0.205) | 0.174 |
| Coma | POM1 vs POD1 | 0.003 (-0.087 to 0.093) | 0.965 | 0.003 (-0.087 to 0.093) | 0.965 |
| DLI | POD1 slope | 0.167 (-1.308 to 1.642) | 0.918 | 0.450 (-1.152 to 2.051) | 0.798 |
| DLI | POW1 vs POD1 | -1.072 (-2.389 to 0.245) | 0.520 | -1.072 (-2.389 to 0.245) | 0.459 |
| DLI | POM1 vs POD1 | 0.362 (-0.955 to 1.679) | 0.853 | 0.362 (-0.955 to 1.679) | 0.798 |
| HO Total | POD1 slope | -0.134 (-0.410 to 0.143) | 0.601 | -0.202 (-0.500 to 0.095) | 0.459 |
| HO Total | POW1 vs POD1 | 0.346 (0.087 to 0.605) | 0.174 | 0.346 (0.087 to 0.605) | 0.174 |
| HO Total | POM1 vs POD1 | 0.063 (-0.196 to 0.322) | 0.856 | 0.063 (-0.196 to 0.322) | 0.804 |
| MTF Cornea Avg Height | POD1 slope | -0.053 (-0.127 to 0.022) | 0.520 | -0.051 (-0.129 to 0.028) | 0.482 |
| MTF Cornea Avg Height | POW1 vs POD1 | 0.074 (-0.021 to 0.170) | 0.520 | 0.074 (-0.021 to 0.170) | 0.459 |
| MTF Cornea Avg Height | POM1 vs POD1 | 0.057 (-0.039 to 0.152) | 0.595 | 0.057 (-0.039 to 0.152) | 0.509 |
| MTF Internal Avg Height | POD1 slope | 0.009 (-0.077 to 0.096) | 0.918 | 0.021 (-0.072 to 0.115) | 0.807 |
| MTF Internal Avg Height | POW1 vs POD1 | -0.036 (-0.114 to 0.042) | 0.601 | -0.036 (-0.114 to 0.042) | 0.578 |
| MTF Internal Avg Height | POM1 vs POD1 | 0.039 (-0.039 to 0.117) | 0.601 | 0.039 (-0.039 to 0.117) | 0.564 |
| MTF Total eye Avg Height | POD1 slope | 0.022 (-0.048 to 0.092) | 0.798 | 0.037 (-0.039 to 0.113) | 0.564 |
| MTF Total eye Avg Height | POW1 vs POD1 | -0.034 (-0.102 to 0.034) | 0.601 | -0.034 (-0.102 to 0.034) | 0.564 |
| MTF Total eye Avg Height | POM1 vs POD1 | -0.001 (-0.069 to 0.067) | 0.970 | -0.001 (-0.069 to 0.067) | 0.970 |
| OSI | POD1 slope | -0.747 (-1.476 to -0.019) | 0.372 | -0.760 (-1.531 to 0.010) | 0.446 |
| OSI | POW1 vs POD1 | 0.625 (-0.292 to 1.542) | 0.520 | 0.625 (-0.292 to 1.542) | 0.459 |
| OSI | POM1 vs POD1 | 0.531 (-0.386 to 1.448) | 0.595 | 0.531 (-0.386 to 1.448) | 0.510 |
| SR Cornea | POD1 slope | -0.070 (-0.161 to 0.021) | 0.520 | -0.069 (-0.166 to 0.028) | 0.459 |
| SR Cornea | POW1 vs POD1 | 0.090 (-0.022 to 0.201) | 0.520 | 0.090 (-0.022 to 0.201) | 0.459 |
| SR Cornea | POM1 vs POD1 | 0.075 (-0.036 to 0.187) | 0.520 | 0.075 (-0.036 to 0.187) | 0.459 |
| SR Internal | POD1 slope | 0.014 (-0.071 to 0.100) | 0.913 | 0.016 (-0.076 to 0.107) | 0.836 |
| SR Internal | POW1 vs POD1 | -0.078 (-0.174 to 0.019) | 0.520 | -0.078 (-0.174 to 0.019) | 0.459 |
| SR Internal | POM1 vs POD1 | -0.016 (-0.113 to 0.080) | 0.913 | -0.016 (-0.113 to 0.080) | 0.836 |
| SR Total eye | POD1 slope | 0.024 (-0.039 to 0.088) | 0.704 | 0.049 (-0.019 to 0.116) | 0.459 |
| SR Total eye | POW1 vs POD1 | -0.048 (-0.116 to 0.020) | 0.520 | -0.048 (-0.116 to 0.020) | 0.459 |
| SR Total eye | POM1 vs POD1 | -0.035 (-0.103 to 0.034) | 0.601 | -0.035 (-0.103 to 0.034) | 0.564 |
| Secondary Astigmatism | POD1 slope | -0.011 (-0.084 to 0.062) | 0.917 | -0.034 (-0.111 to 0.044) | 0.586 |
| Secondary Astigmatism | POW1 vs POD1 | 0.083 (0.007 to 0.159) | 0.336 | 0.083 (0.007 to 0.159) | 0.336 |
| Secondary Astigmatism | POM1 vs POD1 | 0.006 (-0.070 to 0.082) | 0.941 | 0.006 (-0.070 to 0.082) | 0.941 |
| Spherical | POD1 slope | -0.018 (-0.058 to 0.021) | 0.601 | -0.029 (-0.070 to 0.012) | 0.459 |
| Spherical | POW1 vs POD1 | -0.003 (-0.056 to 0.050) | 0.959 | -0.003 (-0.056 to 0.050) | 0.959 |
| Spherical | POM1 vs POD1 | 0.024 (-0.029 to 0.077) | 0.601 | 0.024 (-0.029 to 0.077) | 0.578 |
| Trefoll | POD1 slope | -0.120 (-0.281 to 0.042) | 0.520 | -0.143 (-0.318 to 0.032) | 0.459 |
| Trefoll | POW1 vs POD1 | 0.203 (0.060 to 0.345) | 0.174 | 0.203 (0.060 to 0.345) | 0.174 |
| Trefoll | POM1 vs POD1 | 0.086 (-0.056 to 0.228) | 0.595 | 0.086 (-0.056 to 0.228) | 0.509 |
| The original models were adjusted for age, sex, body mass index, smoking status, diabetes, hypertension, preoperative BCVA, axial length, and corneal astigmatism. The sensitivity models included the same covariates with additional adjustment for marital status and IOL power. HDL-C was modeled as a continuous variable. The POD1 slope represents the adjusted association between HDL-C and each outcome at postoperative day 1, whereas the POW1 vs POD1 and POM1 vs POD1 terms represent changes in the HDL-C slope relative to postoperative day 1. P values were adjusted using the Benjamini–Hochberg false discovery rate method. | | | | | |
|  |  |  |  |  |  |
|  |  |  |  |  |  |
|  |  |  |  |  |  |
|  |  |  |  |  |  |
